# Mice employ a bait-and-switch escape mechanism to de-escalate social conflict

**DOI:** 10.1371/journal.pbio.3002496

**Published:** 2024-10-15

**Authors:** Rachel S. Clein, Megan R. Warren, Joshua P. Neunuebel

**Affiliations:** 1 Department of Psychological and Brain Sciences, University of Delaware, Newark, Delaware, United States of America; 2 Interdisciplinary Neuroscience Program, University of Delaware, Newark, Delaware, United States of America; 3 Data Science Institute, University of Delaware, Newark, Delaware, United States of America; University of St Andrews, UNITED KINGDOM OF GREAT BRITAIN AND NORTHERN IRELAND

## Abstract

Intraspecies aggression has profound ecological and evolutionary consequences, as recipients can suffer injuries, decreases in fitness, and become outcasts from social groups. Although animals implement diverse strategies to avoid hostile confrontations, the extent to which social influences affect escape tactics is unclear. Here, we used computational and machine-learning approaches to analyze complex behavioral interactions as mixed-sex groups of mice, *Mus musculus*, freely interacted. Mice displayed a rich repertoire of behaviors marked by changes in behavioral state, aggressive encounters, and mixed-sex interactions. A distinctive behavioral sequence consistently occurred after aggressive encounters, where males in submissive states quickly approached and transiently interacted with females immediately before the aggressor engaged with the same female. The behavioral sequences were also associated with substantially fewer physical altercations. Furthermore, the male’s behavioral state could be predicted by distinct features of the behavioral sequence, such as kinematics and the latency to and duration of male–female interactions. More broadly, our work revealed an ethologically relevant escape strategy influenced by the presence of females that may serve as a mechanism for de-escalating social conflict and preventing consequential reductions in fitness.

## Abstract 2

Although animals implement diverse strategies to avoid hostile confrontations, the extent to which social influences affect escape tactics is unclear. This study uses innovative computational tools to reveal that male mice avoid conflict by exploiting nearby females to divert the attention of a pursuing aggressor in a "bait-and-switch" strategy.

## Introduction

Social animals navigate complex environments by evaluating sensory cues, assessing risks, integrating new information with existing knowledge, and executing appropriate behaviors [1,2]. This behavioral flexibility is crucial for their physiological fitness, driving the development of cognitive mechanisms to respond to social cues and environmental changes [3]. For example, animals employ transitive inference to deduce social ranks by observing others’ behaviors, enabling them to adapt their actions based on hierarchical status and perceived threats [4]. This adaptability is key to their survival and success.

Animals respond to threats with both learned and innate escape behaviors [5,6] and environmental features significantly influence their choice of escape strategies [7]. For example, associating a neutral context with a noxious stimulus leads to learned freezing behaviors [8], while predator cues or hostile interactions with conspecifics trigger natural, non-conditioned responses [9]. Rodents, for example, adopt a defensive posture and scan their surroundings when they detect predator odors or freeze in response to looming shadows [10,11]. Aggressive conspecifics prompt various escape behaviors depending on the threat’s proximity and the likelihood of evasion [12,13]. Additionally, prior experiences shape escape strategies; animals frequently exposed to conflicts may avoid social encounters to minimize future attacks [14,15]. Whether triggered by pain, predators, or aggression, effectively executing escape strategies is crucial for survival. Failure to do so can lead to significant fitness reductions [6]. Thus, understanding the behavioral strategies animals use to evade danger is essential.

Quantifying and evaluating the effectiveness of naturalistic escape behaviors elicited by hostile interactions is a formidable task. It requires unbiasedly extracting and assessing discrete events within the diverse behavioral repertoires of individual animals. By addressing these challenges, we can gain a comprehensive understanding of the dynamics of escape behavior and the role of behavioral state in this evolutionarily conserved process. In this study, we monitored the behavior of multiple freely interacting mice in a large arena and employed multiple computational approaches to analyze individual behaviors. Using behavioral state as a centralized framework, we discovered a robust phenomenon where males subjected to agonistic encounters appear to escape and avoid conflict by exploiting nearby females to divert the attention of the aggressor. These findings highlight sophisticated social dynamics elucidated through systematic observation of naturalistic behavior, demonstrate the influence of prior social experience and behavioral state on subsequent behavior, and reveal a novel mechanism animals use to escape hostile encounters with aggressive males.

## Results

### Quantifying dynamic social behavior

**Fig 1:**
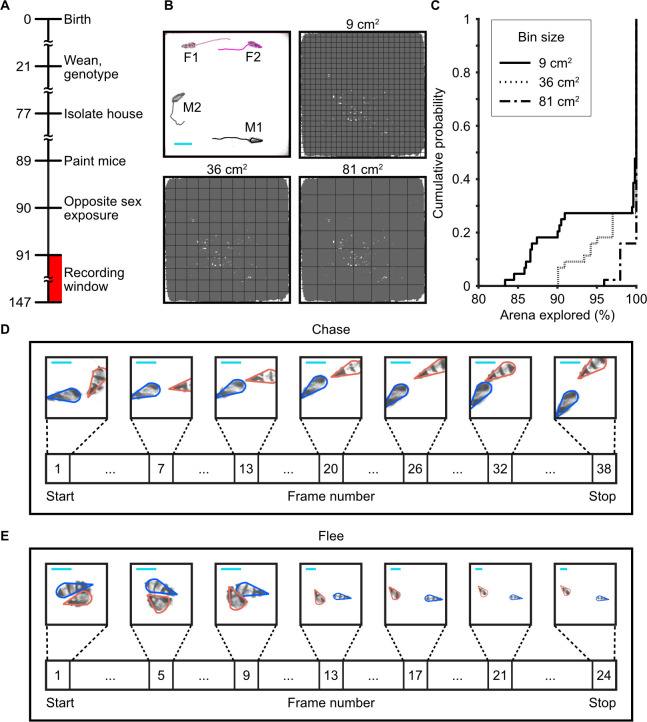
Machine learning-based approaches for tracking individuals and identifying behaviors. (A) Experimental timeline. Numbers denote postnatal day. (B) Individual mice differentiated by distinct fur patterns were tracked in a large arena (width = 76.2 cm, length = 76.2 cm, height = 61 cm) using automated software. Trajectories show 1 s of movement. M1 = male 1, M2 = male 2, F1 = female 1, F2 = Female 2. The scale bar (cyan) represents 10 cm. Trajectory of M2 during the 5-h recording overlaid by grids of 9 cm^2^, 36 cm^2^, and 81 cm^2^. (C) Cumulative probability plots quantifying the percentage of the arena explored by the mice. (D) Chase exemplar. Aggressor males were chasing (outlined in orange), while aggressed males were being chased (outlined in blue). (E) Flee exemplar. Aggressor males were being fled from (outlined in orange), while aggressed males fled (outlined in blue). Numerical values for Fig 1C are available as an online supporting file (S1 Data). Source data can be found in S1–S12 Datasets.

To explore group dynamics, we recorded naturalistic social interactions in mixed-sex groups (*n* = 11, 2 males, 2 females per group) of adult mice for 5 h (**Fig 1A and 1B**). These recordings were previously described in Sangiamo and colleagues [16]. An automated tracking program [17] enabled unbiased quantification of movement (*n* = 44 animals, median total movement = 1,343.7 cm, IQR = 349.3), verifying that each mouse explored the majority of the enclosure (**Figs 1B, 1C and S1**). Tracking information served as input to a supervised machine learning program [18] implemented to identify user-defined innate agonistic behaviors (**Fig 1D**). Agonistic behaviors were defined as events where a male either fled (flee) or was chased (chase) by another male (**Table 1**). Each male exhibited aggressive behavior towards a rival, but in the majority of recordings, one of the males was significantly more aggressive (**S2A–S2D and S3 Figs and S1 Table**). We observed 3,413 agonistic interactions between males (*n* = 22, median = 216, IQR = 331.8), with chasing and flight occurring 2,562 and 851 times, respectively (flight median = 61, IQR = 51.3; chasing median = 174, IQR = 242.3). In these behaviors, each mouse plays a specific, discernable role, revealing 2 distinct behavioral states (aggressor versus aggressed).

**Table 1:** Number and definition of extracted behaviors.

Behavior Name	Behavior Definition	Number of Examples
Chasing	A male follows another male while the 2 mice are within 2 body lengths of each other	2,562
Fleeing	A male running away from the other typically stationary male	851
Walking	A mouse moves around the cage, in isolation (i.e., no other mouse within 35 cm)	21,953
Investigating	Two mice touching, usually including sniffing. Can include nose to body, nose to nose, and/or anogenital investigation. This excludes other defined behaviors.	4,661
Fighting	Both males engaging in physical contact. Involves biting, wrestling, and rolling over each other.	1,177

Interactions between individuals of the opposite sex significantly shape group behavior [19]. To identify social interactions, we developed an automated computational approach (**S2E and S3 Figs**). Social interactions were defined as periods where 2 mice of the opposite sex spent at least 0.2 s within 3 cm of each other. We frequently observed male–female interactions (total interactions = 37,725, median = 3,477, IQR = 742). Despite evidence that females in many species prefer dominant males and acts of aggression serve to attract potential mates [20], the overall aggression level of a male social partner did not influence frequency, overall time, or duration of opposite-sex interactions (**S2E–S2G Fig**).

### Behavioral state directly influences male–female interactions

**Fig 2:**
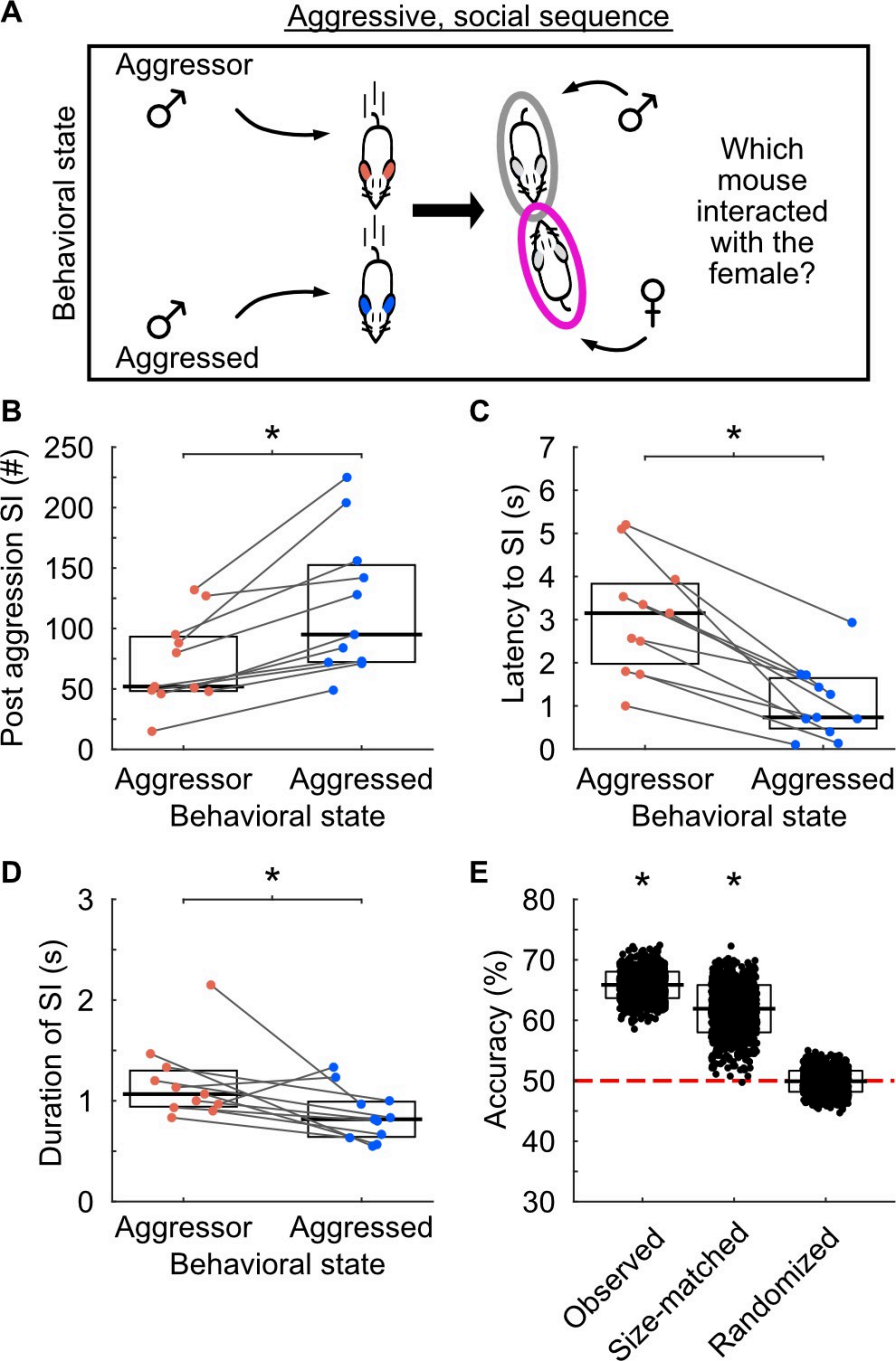
The behavioral state of an individual modulates subsequent interactions with females. (A) Schematic of aggressive social sequences. Sequences consisted of aggressive male interactions followed by male–female social interactions. (B) The number of male–female interactions following aggressive behaviors. Lines connect co-recorded mice. Black lines and white boxes show the medians and interquartile ranges (25%–75%). Each data point represents the median of the distribution for each individual. Wilcoxon signed rank test, W = 0, *p* < 0.001. (C) The latency between aggressive encounters and social interactions. Wilcoxon signed rank test, W = 66, *p* < 0.001. (D) The duration of social interactions following aggressive encounters. Wilcoxon signed rank test, W = 58, *p* < 0.05. (E) Performance of decoders when predicting the behavioral state of the male social partner in post-aggression social interactions. Black lines and white boxes show the means and standard deviations. The red line denotes chance levels (50%). Each condition: 1-sided z-test, *n* = 1,000 iterations. Observed: z = 7.25, *p* < 0.001; size-matched: z = 3.04, *p* = 0.001; randomized: z = −0.05, *p* = 0.48. Numerical values for Fig 2B–2D are available as an online supporting file (S1 Data). Source data can be found in S1–S12 Datasets.

To explore whether a male’s behavioral state affects social engagement, we analyzed the temporal relationship between aggressive behaviors and opposite-sex interactions (**Fig 2A**). Across all recordings, we observed 2,082 sequences where an opposite-sex social interaction followed an aggressive interaction between males. Sixty-two percent of male–female interactions involved aggressed males (**Fig 2B**). The median latency between aggressive behavior and social interaction was 1.17 s (IQR = 4.77 s). However, in 463 sequences, socialization began before the aggressive behavior ended (shortest latency = −7.13 s, median = −0.23 s, IQR = 0.6 s). The delay between hostile male–male interactions and subsequent female interactions was significantly shorter for aggressed males (**Fig 2C**). Additionally, post-aggression interactions were shorter when the aggressed male engaged with the female compared to the aggressor (**Fig 2D**). Decision tree classifiers were used to decode the behavioral state of the male social partner (**Fig 2E**). Accuracies of classifiers, trained on latency to the social interaction and duration of the social interaction, ranged from 58.6% to 72.4%, significantly exceeding chance levels of 50%. When sample-size-matching the number of interactions the aggressor and aggressed had with females, classifiers could predict the behavioral state of the social partner. Randomizing the times of the hostile interactions between males dropped classifier accuracies to chance levels. These results indicate that distinct behavioral states influence subsequent male–female interactions.

We conducted control analyses to determine if these findings were specific to behavioral state. First, we analyzed male–female social interactions following nonaggressive, nonsocial behaviors (e.g., walking, **Table 1 and S4A Fig**), as these sequences differed in the behavioral state and social context. When solitary walks preceded mixed-sex interactions, both the walker and non-walker were equally likely to interact with females (**S4B Fig**). No differences were observed in the latency to interact (**S4C Fig**). The duration of interactions was significantly shorter for non-walking males compared to walking males (**S4D Fig**). Predictive models could differentiate the behavioral state of the male social partner in social interactions following solitary walks, but accuracies dropped to chance levels when controlling for sample size and randomizing behavior times (**S4E Fig**).

Next, we examined a nonaggressive, social behavior (investigating, **Table 1 and S5A Fig**), as these sequences differed in behavioral state but not social context. Similar to walking-triggered sequences, there were no differences in the number of opposite-sex interactions (**S5B Fig**). The latency between investigation and social interaction was shorter when the investigated male was the social partner (**S5C Fig**), but the median latency for investigated males was higher than for aggressed males during aggression-triggered social interactions. The duration of male–female interactions following investigation was indistinguishable by behavioral state (**S5D Fig**). Predictive models failed to accurately identify the behavioral state of the male social partner in social interactions following male–male investigations (**S5E Fig**). These results strongly suggest that aggressive encounters trigger a state-dependent phenomenon, leading to increased interactions between aggressed males and females.

We identified males as more or less aggressive based on aggregated aggression levels (**S2 and S3 Figs**), thus allowing us to assess whether aggressiveness influenced subsequent interactions rather than behavioral state. Comparing more and less aggressive males revealed no differences in the number (**S6B Fig**), latency (**S6C Fig**), or duration of male–female interactions (**S6D Fig**). Predictive models performed at chance levels when identifying the male social partner (**S6E Fig**). These findings suggest that behavioral state, rather than cumulative levels of aggression, underlies the sequential nature of social interactions following aggressive encounters.

### Computational controls

To ensure that sample size did not bias the finding that aggressed males were more likely to engage with females after a hostile interaction (**S7A Fig**), we used a permutation test [21]. We randomly selected 50 sequences from each recording and calculated a difference index between subsequent aggressed and aggressor interactions with a female (**Methods**). For all permutations, the difference index was below zero, suggesting that after hostile interactions between males, the aggressed male consistently interacts with the female first, and the effects are not due to sample size. We employed a similar approach for nonsocial, nonaggressive (**S7B Fig**) and social, nonaggressive (**S7C Fig**) triggered sequences, finding that the distributions of indices were not significantly skewed towards either male. These analyses suggest that neither sample size nor a subset of examples underlies the results.

To further address the importance of behavioral state, we performed another permutation analysis. First, we randomized the identity of the males during aggressive encounters that preceded social interactions. We then calculated a difference index between subsequent aggressed and aggressor interactions with the shuffled data. This procedure was performed 1,000 times to generate a distribution of index values. The observed difference index (−0.25) was significantly lower than the mean of the distribution of shuffled index values (**S7D Fig**). Additionally, decision tree classifiers were used to predict the type of behavioral sequences (aggressive or control behavior—combined walking and investigating) at rates higher than chance levels (**S7E Fig**). The model’s accuracy did not depend on the number of examples, maintaining high accuracy with equal numbers of aggressive and control sequences (**S7E Fig**). Accuracy was low when we attempted to decode sequence type on data where the timing of the behavior preceding the social interaction was randomized (**S7E Fig**). Together, these analyses substantiate the finding that a male’s behavioral state affects subsequent interactions with females after a hostile interaction.

### Behavioral controls

**Fig 3:**
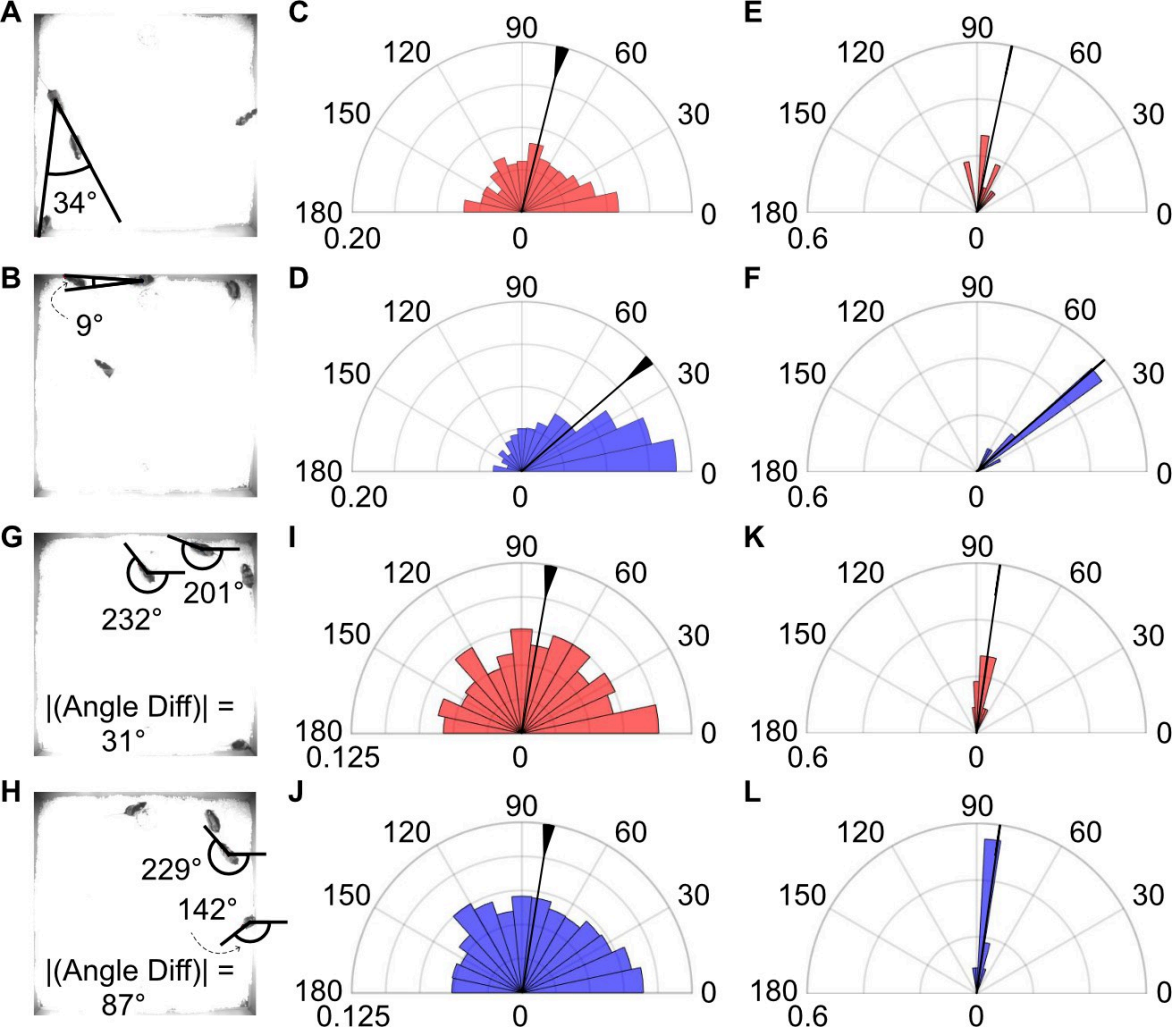
State and orientation of aggressed male influence aggression-triggered social interactions, independent of female’s orientation. (A) Example showing the angle between the aggressor male’s heading direction and the vector pointing from the male to the female at the end of an aggressive encounter. (B) As in B, for the aggressed male. (C) Distribution of angles between the aggressor male’s heading direction and the vector pointing from the male to the female at the end of the aggressive encounter. Theta and radians on the polar plot represent angles in degrees and normalized frequency. Normalization was determined by dividing the number of occurrences in each bin by the number of post-aggression interactions between the aggressor and female. Circular variation and median shown by triangle and line. (D) As in C, for the aggressed male. Comparing aggressive and aggressed state: Watson’s U2, U2 = 2.12, *p* < 0.001. (E) Median angle between the aggressor’s heading direction and the vector pointing from the animal to the female for individuals. (F) As in E, for the aggressed male. Comparing aggressive and aggressed state for individuals: Watson’s U2, U2 = 0.34, *p* < 0.005. (G) Example showing the angle difference between the orientations of aggressor male and female social partner. (H) As in G, for the aggressed male. (I) Distribution of angle differences at the end of the aggressive behaviors when aggressor was the social partner. (J) As in I, for the aggressed male. Comparing aggressive and aggressed state: Watson’s U2, U2 = 0.05, *p* = 0.81. (K) Median angle difference between the orientations of aggressor male and female social partner for individuals. (L) As in K, for the aggressed male. Comparing aggressive and aggressed state for individuals: Watson’s U2, U2 = 0.06, *p* = 0.57. Numerical values for Fig 3C, 3D, 3I, 3J, 3E, 3F, 3K and 3L are available as an online supporting file (S1 Data). Source data can be found in S1–S12 Datasets.

Differences in male trajectories following aggressive behaviors might drive the observed behavioral patterns. To investigate how trajectory differences might influence subsequent social interactions, we quantified the angles between the male’s heading direction and the vector pointing from the male to the female (**Fig 3A and 3B**). Across all sequences, we found that, on average, the angle difference was significantly smaller for aggressed animals (**Fig 3C and 3D**). This trend persisted when examining the medians of individual animals (**Fig 3E and 3F**). To assess whether these differences were influenced by the orientation of the females relative to the males, we calculated the difference in male and female heading direction at the end of the aggressive behavior (**Fig 3G and 3H**). There were no significant differences between aggressor and aggressed males, indicating that the orientation of the females did not affect the likelihood of a particular male engaging in subsequent interactions (**Fig 3I–3L**). These results suggest that the heading direction of the aggressed male, but not the orientation of the female, plays a significant role in subsequent interactions.

**Fig 4:**
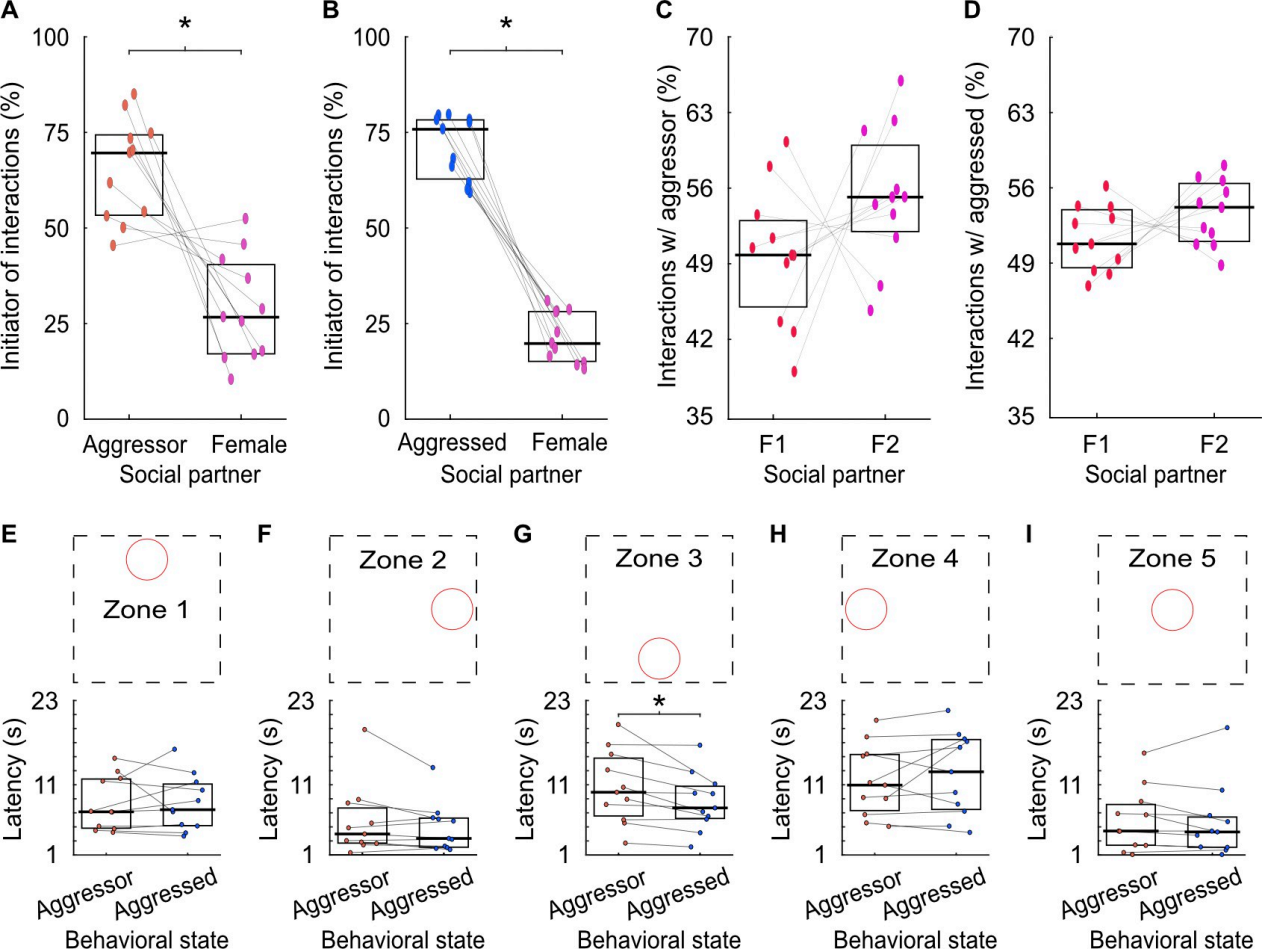
State-dependent aggression-triggered social interactions are independent of initiator or location in cage. (A) Male–female social interactions initiated by the aggressor or female. Lines connect co-recorded mice. The horizontal bars and boxes below the data show the medians and interquartile ranges (25%–75%). Wilcoxon signed rank test, W = 64, *p* < 0.005. (B) As in A, for interactions with the aggressed. Wilcoxon signed rank test, W = 69, *p* < 0.001. (C) Interactions the aggressor male had with each female. Wilcoxon signed rank test, W = 16, *p* = 0.15. (D) As in C, for interactions with the aggressed. Wilcoxon signed rank test, W = 20, *p* = 0.08. (E) Top: Schematic showing arbitrary zone in the cage. Bottom: Median latency of aggressor and aggressed to reach zone 1 from the end of all aggressive behaviors. Wilcoxon signed rank test, W = 32, *p* = 0.98. (F) As in E, for zone 2. Wilcoxon signed rank test, W = 47, *p* = 0.24. (G) As in E, for zone 3. Wilcoxon signed rank test, W = 64, *p* < 0.05 (α set to 0.01—Bonferroni correction to control for *familywise* error rate and correct for multiple comparisons). (H) As in E, for zone 4. Wilcoxon signed rank test, W = 23, *p* = 0.41. (I) As in E, for zone 5. Wilcoxon signed rank test, W = 32, *p* = 0.30. Numerical values for Fig 4A–4I are available as an online supporting file (S1 Data). Source data can be found in S1–S12 Datasets.

Despite the aggressed male’s orientation towards the female at the end of aggressive encounters, it is possible that females initiate the subsequent social interaction. We measured the instantaneous speeds and positions of each social partner to identify the initiator of male–female interactions following aggressive encounters. Males consistently initiated opposite-sex social interactions regardless of behavioral state (**Fig 4A and 4B**). This phenomenon was consistent across females, as males in both behavioral states engaged in post-aggression social interactions with each female (**Fig 4C and 4D**). These results indicate that the onset of social encounters following aggressive behavior is driven by the aggressed males rather than by female social partners.

To rule out the possibility that the aggressed animal encounters the female by chance while escaping from the aggressor, we quantified the latency to reach multiple arbitrary zones, or spatial locations, in the behavioral arena after the aggressive behavior ended. The zones included the North central (zone 1), East central (zone 2), South central (zone 3), West central (zone 4), and center (zone 5) areas of the cage. When controlling for *familywise* error rate and correcting for multiple comparisons using a Bonferroni correction, we found no differences in latency to reach any of the zones except for zone 3 (**Fig 4E–4I**), supporting our hypothesis that this phenomenon is state dependent. Overall, these results demonstrate that the heading direction and subsequent actions of the aggressed male play a critical role in initiating post-aggression social interactions, and that this behavior is not driven by the orientation or actions of the females.

### Temporal persistence of post-aggression social interactions

**Fig 5:**
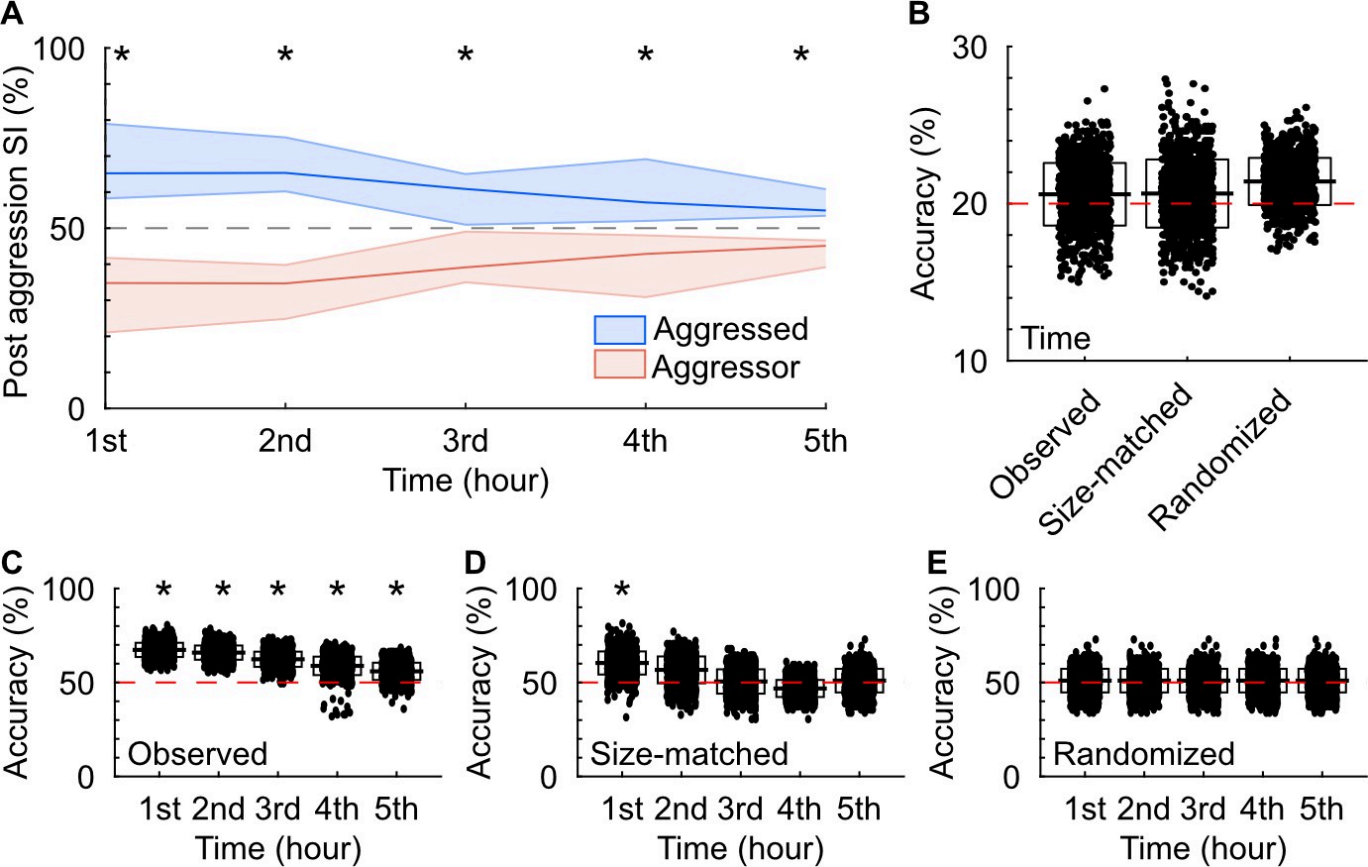
Aggression-triggered female social interaction sequences emerge early and persist over time. (A) The percentage of aggression-triggered sequences binned hourly for individuals in either an aggressed or aggressive behavioral state. The lines and shaded regions show the medians and interquartile ranges (25%–75%). Aggressor and aggressed compared each hour with Wilcoxon signed rank test. Hour 1: W = 2, *p* < 0.005; hour 2: W = 2, *p* < 0.005; hour 3: W = 3.5, *p* < 0.05; hour 4: W = 4.5, *p* < 0.05; hour 5: W = 1, *p* < 0.05. (B) Decoders’ performance when predicting when the sequences occurred. The horizontal bars and boxes below the data show the means and standard deviations. The red line denotes chance levels. Each condition: 1-sided z-test, *n* = 1,000 iterations. Observed: z = 0.30, *p* = 0.38 size-matched: z = 0.30, *p* = 0.38 randomized: z = 0.94, *p* = 0.17. (C) Decoders’ performance when predicting the behavioral state of the male interacting with the female for all observed data. Accuracy compared to chance each hour with 1-sided z-test, *n* = 1,000 iterations. Hour 1: z = 4.6, *p* < 0.001; hour 2: z = 4.2, *p* < 0.001; hour 3: z = 3.1, *p* = 0.001; hour 4: z = 1.8, *p* = 0.04; hour 5: z = 1.7, *p* = 0.04. (D) As in C, for size-matched controls. Accuracy compared to chance each hour with 1-sided z-test, *n* = 1,000 iterations. Hour 1: z = 1.7, *p* = 0.04; hour 2: z = 0.9, *p* = 0.13; hour 3: z = 0.1, *p* = 0.47; hour 4: z = −0.7, *p* = 0.24; hour 5: z = 0.2, *p* = 0.44. (E) As in C, for randomized start times of aggressive male–male interactions. Accuracy compared to chance each hour with 1-sided z-test, *n* = 1,000 iterations. Hour 1: z = 0.39, *p* = 0.35; hour 2: z = 0.26, *p* = 0.36; hour 3: z = 0.30, *p* = 0.38; hour 4: z = 0.34, *p* = 0.37; hour 5: z = 0.63, *p* = 0.26. Numerical values for Fig 5A are available as an online supporting file (S1 Data). Source data can be found in S1–S12 Datasets.

Aggression-triggered social interaction sequences emerged early and persisted over time. The initial sequence in each recording occurred at 10.26 min on average (*n* = 11, median = 5.82 min, IQR = 3.78 min), whereas the final event was recorded at 283.24 min on average (median = 297.27 min, IQR = 170.20 min). Throughout the 5-h recordings, behavioral state significantly influenced these interactions, with aggressed males engaging in a higher proportion of aggression-triggered interactions during each hour (**Fig 5A**). Multi-class support vector machines (mcSVMs) trained on behavioral sequence features failed to accurately predict the specific hour of occurrence (**Fig 5B**). Conversely, decision tree classifiers effectively predicted the behavioral state during each hour (**Fig 5C**). However, when controlling for the number of sequences per hour, model accuracy was significantly above chance only during the first hour (**Fig 5D**). Randomizing the hour of sequence occurrence resulted in model accuracies indistinguishable from chance (**Fig 5E**), indicating consistent features in aggression-triggered sequences over time. These findings suggest that the aggressive behavioral state is a key mediator of subsequent social interactions throughout the experiments.

### Escape mechanism for deescalating hostile confrontations

**Fig 6:**
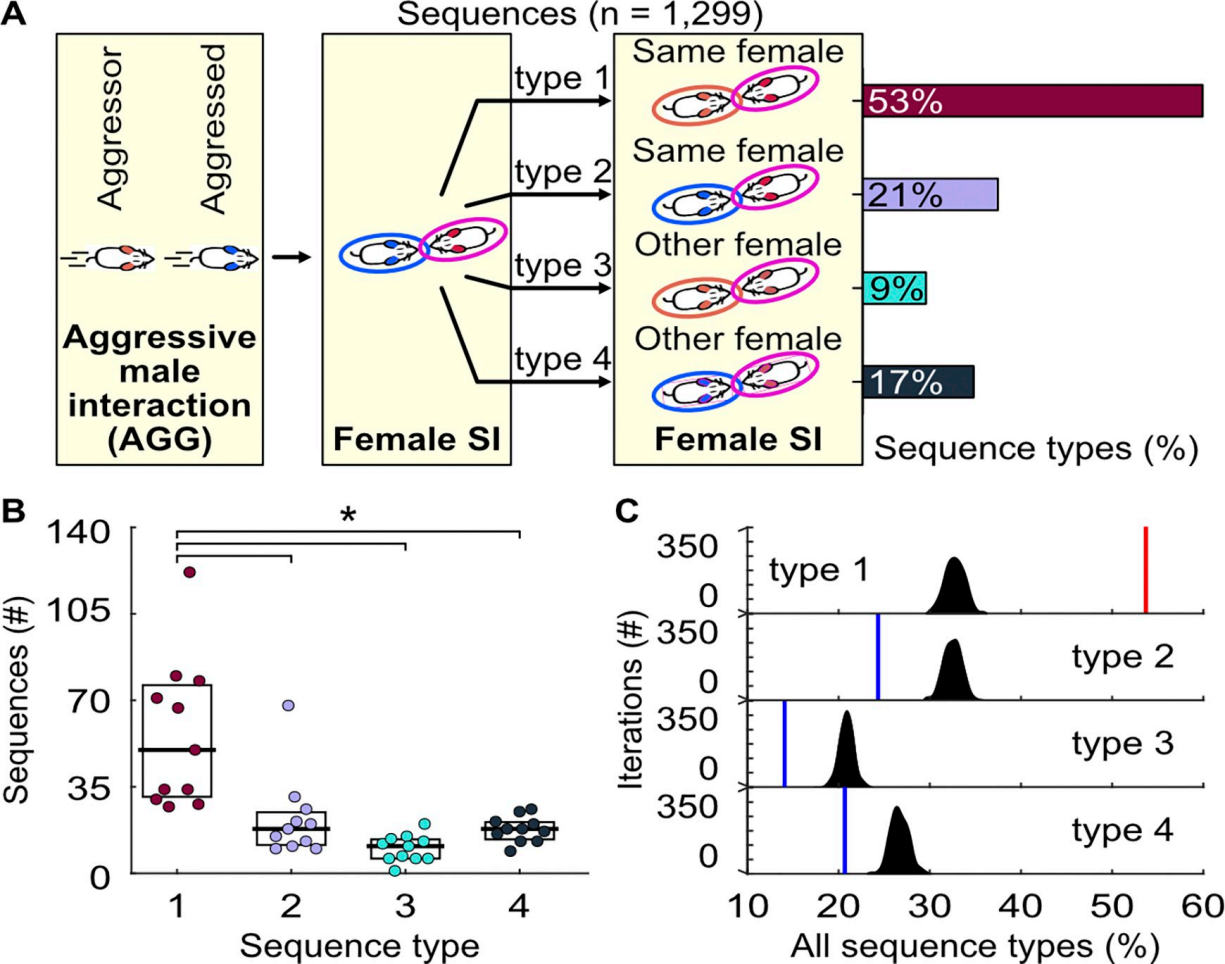
Quantification of sequential behaviors after aggressive encounters. (A) Schematic of sequence types and frequency of occurrence. (B) The number of each sequence type across recordings. The horizontal bars and boxes below the data show the medians and interquartile ranges (25%–75%). Kruskal–Wallis test (H(3,40) = 19.93, *p* < 0.001) with Dunn–Sidak correction. Type 1 vs. 2: *p* < 0.001; type 1 vs. 3: *p* < 0.001; type 1 vs. 4: *p* < 0.001. (C) Difference between the percentage of sequence types and randomized distributions of sequences. Red and blue vertical lines denote percentages significantly above or below chance. Actual proportions compared to randomized data with 2-sided z-test, *n* = 1,000 iterations. Type 1: z = 20, *p* < 0.001; type 2: z = −7.4, *p* < 0.001; type 3: z = −7.6, *p* < 0.001; type 4: z = −5.9, *p* < 0.001. Numerical values for Fig 6B are available as an online supporting file (S1 Data). Source data can be found in S1–S12 Datasets.

Animals employ various strategies to avoid confrontations, as injury reduces biological fitness [6, 21]. We hypothesized that aggressed males might interact with a female to divert the attention of an aggressor. To test this possibility, we examined social interactions that followed the interactions after aggressive encounters (**Table 2**). We observed that the aggressor often engaged with the same female the aggressed male interacted with (**S1 Video**). Specifically, the majority of subsequent interactions occurred between the aggressor and the same female (**Fig 6A**, Sequence type 1, 53%). Other interaction sequences included the aggressed male interacting with the same female (Sequence type 2, 21%), the aggressor with a different female (Sequence type 3, 9%), and the aggressed male with a different female (Sequence type 4, 17%). Sequence type 1 occurred significantly more often than the other types (**Fig 6B**) and more frequently than expected by chance (**Fig 6C**). When training an mcSVM to classify sequence types based on speed and distance traveled during the aggressive encounter, we found no distinction among the sequences (**Methods**; mean accuracy = 27%, standard deviation = 3%, 1-sided z-test, *n* = 1,000 iterations, z = 0.79, *p* = 0.43), demonstrating that the aggressive states initiating these sequences are similar. Together, these findings suggest that aggressed males may use a bait-and-switch tactic to evade aggressors.

**Table 2:** Description of sequence types.

Type	Behavior One	Behavior Two	Behavior Three
1	Aggressive encounter	Social interaction (aggressed and female)	Social interaction (aggressor and same female)
2	Aggressive encounter	Social interaction (aggressor and female)	Social interaction (aggressed and same female)
3	Aggressive encounter	Social interaction (aggressed and female)	Social interaction (aggressor and other female)
4	Aggressive encounter	Social interaction (aggressor and female)	Social interaction (aggressed and other female)

**Fig 7:**
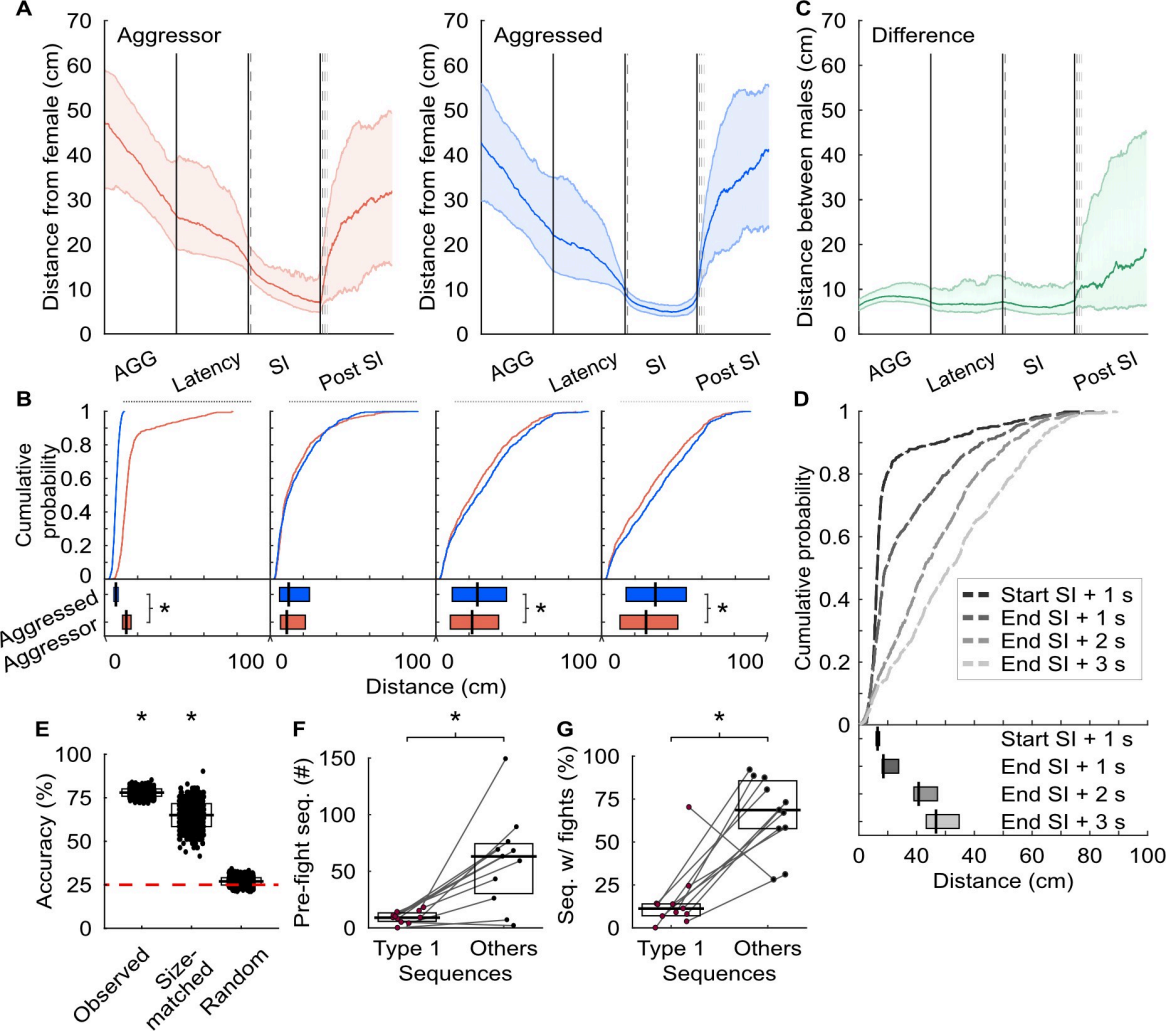
Submissive state-dependent behavioral strategies after aggressive encounters. (A) For sequence type 1, distances between the aggressive or aggressed males and the female social partner were calculated during aggressive behaviors (AGG), the time between AGG and male–female social interactions, interactions, and 5 s post-interaction. The lines and shaded regions show the medians and interquartile ranges (25%–75%). The dashed lines indicate times representing 1 s after the start of the social interaction and post-interaction times of 1, 2, and 3 s. (B) For sequence type 1, quantification of distances 1 s after the start of the social interaction and post-interaction times of 1, 2, and 3 s. Times correspond to the colored dashed lines in A. Top: all distances. Bottom: median distances for each mouse and compared using Wilcoxon signed rank test. start + 1 second: W = 192,814, *p* < 0.0001; end + 1 second: W = 71,772, *p* < 0.0001; end + 2 second: W = 73,311, *p* < 0.0001; end + 3 second: W = 79,583, *p* < 0.0001. (C) As in A, but measuring distance between males. (D) As in B, but measuring distance between males. Top: all distances. Bottom: median distances for each mouse compared using Kruskal–Wallis test (H(3,40) = 34.13, *p* < 0.0001) with Dunn–Sidak correction. start of SI + 1 second vs. end of SI + 2 seconds: *p* < 0.001; start of SI + 1 second vs. end of SI + 3 seconds: *p* < 0.0001; end of SI + 1 second vs. end of SI + 3 seconds: *p* < 0.005. (E) Decoders’ performance predicting sequence type. The horizontal bars and boxes below the data show the means and standard deviations. The red line denotes chance levels. Each condition: 1-sided z-test, *n* = 1,000 iterations. Observed: z = 25.0, *p* < 0.001; size-matched: z = 6.06, *p* = 0.001; randomized: z = 0.93, *p* = 0.18. (F) Fights occurring in the presence of type 1 sequences or other sequence types. Lines connect co-recorded mice. Wilcoxon signed rank test, W = 1, *p* < 0.005. (G) Percentage of sequences with fights. Lines connect co-recorded mice. Wilcoxon signed ranked test, W = 3, *p* < 0.01. Numerical values for Fig 7B and 7D–7G are available as an online supporting file (S1 Data). Source data can be found in S1–S12 Datasets.

To further investigate this tactic, we analyzed the distances between the female and both males at various time points: during the aggressive encounter, the period between the encounter and the first social interaction, the first social interaction, and the subsequent 5 s (**Figs 7A, 7B and S8**). In all sequence types, the aggressed male was significantly closer to the aggressor one second after the social interaction began. However, in sequence type 1, distances between both males and the female were comparable one second after the interaction ended, with the aggressed male then moving farther away from the female at subsequent time points. This pattern was unique to type 1 sequences, indicating a specific escape strategy. Furthermore, for sequence type 1, the distance between males increased after the second interaction (**Fig 7C and 7D**), suggesting that the interaction disrupts the aggression sequence.

We also examined whether kinematic patterns could predict sequence types. Using mcSVMs, we found that distances at 6 key time points could decode sequence type (**Fig 7E**). To control for bias due to the high number of type 1 sequences, we size-matched the sequence types and still found better-than-chance classification (**Fig 7E**). Randomizing sequence type labels did not yield significant results (**Fig 7E**). These results strongly support the hypothesis that aggressed males use a bait-and-switch tactic.

Escaping aggressive conspecifics and avoiding costly encounters is advantageous to an individual’s well-being [3]. If aggressed animals successfully use a bait-and-switch like mechanism to escape hostile interactions and de-escalate social conflict, then fewer fights should occur between male interactions. Alternatively, the bait-and-switch could aggravate the aggressor and trigger an aggressive response, thus increasing the number of fights and escalating costly social conflicts. To address these 2 possibilities, we trained a supervised machine-learning classifier to detect fights (**Table 1**). There were 1,177 fights detected across all recordings (median = 88, IQR = 63.75); however, fights rarely occurred after type 1 sequences (**Fig 7F**). Additionally, the proportion of fights following a bait-and-switch was significantly lower compared to other sequences (**Fig 7G**). Our findings suggest the bait-and-switch sequence helps animals avoid further aggression and de-escalates conflicts.

## Discussion

Animals continually observe and adjust to changes in their social environment, integrating sensory feedback, previous social experiences, and internal states to modify their behavior [3]. In this study, we used sophisticated, unbiased methods to quantify the behavior of males in groups of freely behaving mice (**Figs 1 and S1–S3**), establishing aggressor-aggressed behavioral states as a framework to examine natural social dynamics. We found that males employ behavioral-state-dependent strategies after hostile interactions to evade aggressors and de-escalate confrontations. Specifically, we observed that aggressed males were more likely to interact with a female immediately following antagonistic encounters (**Fig 2**). These brief post-aggressive interactions occurred frequently and consistently throughout the five-hour recordings, indicating the robustness of this behavior (**Fig 5**). Most subsequent male–female interactions involved the female previously engaged by the aggressed male and the aggressor, suggesting a bait-and-switch strategy (**Figs 6 and 7**). This strategy appears to mitigate the costs of agonistic encounters, as fights rarely occurred following bait-and-switch sequences (**Figs 6 and 7**).

Animals use a diverse repertoire of defensive actions to escape threats [6,9,22–24]. While environmental cues are crucial for optimizing defense against predation, animals must also assess trade-offs, as escaping can result in a loss of resources, such as territory or mating opportunities [22]. In our study, we observed that aggressed males sacrifice additional time with females, potentially reducing their chances to copulate in favor of avoiding hostile interactions. We found that aggressed males—not females—initiate social interactions after aggressive encounters but then quickly move away as the aggressor approaches the female (**Figs 4, 6 and 7**). This movement pattern suggests that aggressed males may use a bait-and-switch strategy to distract the aggressor with a potential mating opportunity. This tactic reflects the ability of animals to incorporate information about their surroundings and make optimal choices to avoid threats. These findings indicate that mice may integrate information about their own behavioral state and that of others to shape dynamic social interactions and implement effective behavioral strategies.

Hierarchical rank or social status regulates interactions across many species, from wasps and fish to humans and primates [25,26]. Once a hierarchical rank is established, the frequency of switching between aggressor and aggressed roles decreases [27]. In such hierarchies, dominant animals often act aggressively to reinforce their status [28]. These interactions follow a predictable pattern: dominant animals initiate with threat displays, escalating to physical attacks if necessary [29–31]. Submissive animals respond by detecting the threat, initiating an escape response, and terminating it once safe [6,11]. Social status can influence these escape strategies [15,32], as seen in crayfish [33], where changes in rank alter avoidance behaviors during aggressive encounters. Furthermore, social rank impacts responses to chronic psychosocial stress. For example, Larrieu and colleagues [34] found that dominant mice develop avoidant behaviors after repeated defeats, indicating that past experiences shape behavioral strategies. Although we are unable to directly measure hierarchical rank in these experiments, our findings suggest that the behavioral state of an animal, rather than the overall level of aggression, influences the bait and switch escape strategy.

Our findings indicate that males engage in specific behavioral patterns after aggressive encounters, which are associated with a reduction in subsequent fights. We interpret this as a potential bait-and-switch strategy used by males in a submissive state to de-escalate confrontations. However, our experimental design, aimed at analyzing naturalistic behavior, limited our ability to thoroughly investigate the mechanisms underlying this phenomenon. This study primarily describes a frequently occurring behavior in males and provides the computational tools needed for future research to explore the mechanistic aspects of this potential escape strategy. Our study utilized small, sex-balanced groups, which may not accurately represent natural conditions outside the laboratory. Future research should vary the number of animals, combinations of males and females, and environmental factors to better understand the applicability of this strategy in more naturalistic settings. Additionally, our focus on animals without a clear social hierarchy may have influenced the observed behaviors. Social status significantly affects behavioral decisions [35–37], and animals with established social ranks might exhibit different behavioral strategies. Here, we characterized a behavioral sequence in which the female acts as a more potent distractor, taking precedence over the aggressed male. Several questions remain, such as whether males can distract aggressors with objects in the environment or female scent cues. Given that females also form social hierarchies and employ strategies to evade unwanted male attention [38–40], it is possible that female mice use similar strategies to escape aggressive interactions. Future experiments should explore female behavior to determine if this phenomenon is also present. Despite the limitations of our study, we identified a discrete, state-dependent behavioral strategy in which animals use social information to guide their actions. This bait-and-switch strategy provides a framework for studying flexible, socially relevant behaviors in laboratory settings and investigating the underlying neural mechanisms using a powerful suite of readily available computational tools.

## Methods

### Experimental model and subject details

We used adult (13 to 21 weeks) male (*n* = 22) and female (*n* = 22) B6.CAST-Cdh23Ahl+/Kjn mice (Jackson Laboratory, Bar Harbor, Maine, United States of America, stock: 002756). This congenic mouse strain, which is less susceptible to age-related hearing loss, was selected as our previous study aimed to investigate the relationship between ultrasonic vocalizations and social behavior [16].

Mice were housed in a humidity-controlled, temperature-regulated colony room at the University of Delaware on a 12-h light/dark cycle (lights off at 7:00 PM EST). At 3 weeks old, mice were weaned and genotyped using tail samples sent to TransnetYX (Cordova, Tennessee, USA), ensuring only Cdh23-expressing mice were used in behavioral experiments. Mice were individually tagged with light-activated microtransponders (p-Chip, PharmaSeq) implanted subcutaneously at the base of the tail. Post-weaning, mice were group-housed with same-sex siblings (3 to 5 per cage), with all cages containing ALPHA-dri bedding and environmental enrichment. Mice had ad libitum access to food and water.

### Ethics statement

The University of Delaware’s Institutional Animal Care and Use Committee (IACUC), adhering to National Institutes of Health standards, approved all experimental protocols (AUP Numbers: 1275-2014-0, 1275-2017-0, 1275-2020-0).

### Software and algorithms

Matlab 2013, Matlab 2014, Matlab 2016

MOuse TRacker (MOTR, https://motr.janelia.org) [17]

Janelia Automatic Animal Behavior Annotator (JAABA, https://jaaba.sourceforge.net) [18]

### General experimental design

At least 2 weeks before the behavioral experiment, size-matched (male with male and female with female) mice were housed individually to minimize the effects of group housing and hierarchical rank on social behavior [41,42]. For identification, mice were marked with unique back patterns using nontoxic hair dye (Clairol Nice ‘N Easy, Born Blonde Maxi) under light anesthesia at least 2 days before recording [17]. Each mouse received a random pattern: 5 dots, 1 diagonal slash, 2 vertical lines, or 2 horizontal lines. The day after marking, mice underwent a 10-min exposure to a mouse of the opposite sex to enhance social communication [43,44]. The opposite-sex partners were not used in behavioral recordings but were reused with multiple test subjects. If copulation attempts occurred, a trained observer ended the session. Two hours before recording, the estrous stage of female mice was assessed using noninvasive vaginal lavage and cytological analysis [45,46]. Cells were collected with a saline wash, placed on a slide, stained with crystal violet, and examined under a light microscope (VWR, 89404–890). Photographs were taken using a camera attached to the microscope (World Precision Instruments, USBCAM50 and 501381). Females were considered in estrus if their cells were predominantly cornified squamous epithelial cells lacking a nucleus. Recordings proceeded only if both females were in estrus; otherwise, estrous testing continued until both were in estrus. We used females in estrus because this stage of the reproductive cycle induces male competition and, consequently, a more diverse behavioral repertoire is observed [47–49].

For each recording, 2 male and 2 female mice were housed together for 5 h in a mesh-walled cage (McMaster-Carr, 9218T25) lined with Sonex foam (VLW-35, Pinta Acoustic), ensuring sufficient behavioral sampling for temporal analysis. The cage was placed in a custom-built anechoic chamber. Three of the 11 recordings used a cylindrical cage (height: 91.4 cm; diameter: 68.6 cm), while the remaining 8 used a cuboid cage with an extruded aluminum frame (8020) (width: 76.2 cm; length: 76.2 cm; height: 61.0 cm) (**S1 Fig**).

Video data were continuously recorded at 30 frames per second using a camera (GS3-U3-41C6M-C, FLIR) controlled and synchronized by custom written software. Data were stored on a PC (Z620, Hewlett-Packard). Infrared lights (IR-LT30, GANZ) were positioned above the cage to illuminate the arena for mouse tracking. ALPHA-dri bedding was added to the cage to a depth of approximately 0.5 inches to enhance color contrast between the cage floor and the mice. Each mouse was recorded individually for 10 min post-experiment to facilitate automated tracking [17].

### Data processing

We analyzed mouse trajectories using a data analysis pipeline on the University of Delaware’s FARBER computer cluster (http://docs.hpc.udel.edu). The program MOTR was employed to track the mice by fitting an ellipse around each mouse in every video frame. MOTR calculated the x and y coordinates of the ellipse’s center, its orientation, and the lengths of the semi-major and semi-minor axes. Additionally, the nose position, distance from other animals, and instantaneous speed for each mouse at every frame was determined. A trained observer visually inspected the trajectories after tracking to ensure accuracy.

### Quantifying exploration

To assess exploration (**Fig 1C**), we divided the arena into evenly spaced bins of 3 sizes: 9 cm^2^, 36 cm^2^, and 81 cm^2^. For each animal, we calculated the percentage of bins explored during the experiment.

### Automatic extraction of social behaviors

We utilized JAABA, a supervised machine-learning program, to extract behaviors (**Table 3**) based on definitions from prior research [16]. We focused on behaviors with clearly identifiable states: (1) aggressive, social behaviors were chasing and fleeing; (2) nonaggressive, nonsocial behavior was walking; and (3) nonaggressive, social behavior included male–male investigation. Behavioral states were categorized as follows: (1) aggressor (the chasing male or the one being fled from) and aggressed (the chased male or the one fleeing) for aggressive social behaviors; (2) walking or not walking for nonaggressive nonsocial behaviors; and (3) investigating and being investigated for nonaggressive social behaviors. Fights were extracted but not included in social aggressive behaviors, as our focus was specifically on assessing behavioral roles, and assigning roles was ambiguous at best during fights. All behaviors exhibited a false positive rate below 5%, as determined by manual ground truthing [16]. To gauge differences in aggression across recordings (**S2A–S2D Fig**), we computed an aggression score. This score was derived by subtracting the number of aggressive behaviors performed by male one from those performed by male two, then dividing by the total number of aggressive behaviors. In each recording, we determined which male was more or less aggressive by comparing the number of aggressive behaviors, assuming equal expression of aggression.

**Table 3:** Creating JAABA classifiers.

Behavior Name	Behavior Definition	Post Hoc Refinements
Male chase male	A male follows another male while the 2 mice are within 2 body lengths of each other	Duration >6 frames Trajectories of males overlapping by at least 20% Confidence scores >0.5 Distance between males <30 cm Distance between chased animal and closed female >15 cm
Male being chased	The male that is being followed	Same as male chase male
Flee	A male running away from the other male	Duration >10 frames Confidence score >0.7 Closest animal <8cm
Fled from	The male that the fleeing male is escaping; this male is typically stationary	Same as flee
Walk	A mouse moves around the cage, in isolation (i.e., no other mouse within 35 cm)	Duration >20 frames Average score >1
Male investigate male	Two mice touching, usually including sniffing. Can include nose to body, nose to nose, and/or anogenital investigation. This excludes other defined behaviors.	Confidence score >0.11 Speed <0.19 and >0.015 Average distance between males >0.04 cm
Fight	Both males engaging in physical contact. Involves biting, wrestling, and rolling over each other.	Duration >30 frames Confidence score >1.5 Distance between males and nearest female at start >5 cm Average speed >7.5

### Quantifying social interaction

We employed a custom-written Matlab script to quantify social interaction. Mice pairs were considered socially interacting when their separation was less than one body length (≤6 cm). To accomplish this, we utilized the mouse’s centroid position, major axis, minor axis, and heading direction to automatically fit a social ellipse around each animal. These ellipses encompassed each of the 4 animals in every video frame, each extending 3 cm in front and behind the animal. Social interaction was defined as periods when ellipses overlapped (**S2E–S2G Fig**). To distinguish social interactions from brief encounters, interactions were required to last at least 6 frames. This threshold was determined via manual inspection.

### Sequences of behavior

#### Aggressive social behaviors

To examine the dynamics between aggressive behaviors and male–female social interactions (**Fig 2A–2D**), we identified the start and end times of the aggressive behaviors and the actors involved. We organized the behaviors temporally to identify sequences where a male–female social interaction followed an aggressive behavior, termed aggression-triggered SIs. We quantified the number of times males in either behavioral state participated in these SIs.

The latency between aggressive behavior and SI was calculated by subtracting the SI start time from the aggressive behavior end time, including cases where the SI began before the aggressive behavior ended. SI duration was determined by subtracting the end time from the start time. For each recording, we calculated the median SI duration and latency for both aggressors and aggressed individuals. We also analyzed the number of sequences, median latency, and median duration using aggregate aggression levels to assess the role of hierarchical rank (**S6A–S6D Fig**).

We compared the angles between the male’s heading direction and the vector from the male to the female (**Fig 3A–3F**) using coordinates from MOTR. The male’s heading direction vector (v1) was determined from the male’s body center and nose, while the vector to the female (v2) was from the male’s body center to the female’s body center. The angle between vectors was calculated using the following:

theta=atan2(norm(det([v2;v1])),dot(v1,v2)),

where the Matlab functions atan2, norm, det, and dot represent the four-quadrant inverse tangent, vector normalization, matrix determinant, and the dot product, respectively.

We also used the Matlab function angdiff to calculate the difference in ellipse angles between social partners (aggressor/aggressed and female) (**Fig 3G–3L**), with statistical differences assessed via Watson’s U2 test (Matlab File Exchange). Circular median and variance were calculated using the Matlab circular statistics toolbox.

When determining interaction initiators, we analyzed the animals’ instantaneous speeds and their physical positions relative to each other. Instances of stationary behavior, such as grooming, were identified when the instantaneous speed fell below 0.023 cm/second. Mice exhibiting such speeds at the onset of social interactions were categorized as stationary. Typically, interactions were initiated by the nonstationary mouse when one mouse was stationary. Thus, in interacting pairs with one stationary mouse, the other mouse was classified as the initiator. In cases where both mice had speeds exceeding 0.023 cm/s, we determined the initiator by assessing when the front of their ellipses began to overlap. Nose position and ellipse orientation were utilized to determine heading direction. If both animals were heading toward each other and their ellipses overlapped within 6 frames, the initiation was classified as mutual. To validate the accuracy of our initiation calculations, the results were compared with those of a trained human observer. Results indicating the initiator of social interactions are depicted in **Fig 4A–4D**.

We quantified the latency for males to reach various cage zones after aggressive behavior (**Fig 4E–4I**), with circles of 6 cm radius at 5 locations. Latency values were averaged and compared between aggressor and aggressed.

#### Nonaggressive, nonsocial behaviors

We assessed the relationship between nonsocial behaviors and SIs by repeating the aggression-triggered SI analyses using walking-triggered SIs (**S4A–S4D Fig**). The number of aggressive behaviors was sample-size-matched by randomly selecting a subset of walks. We calculated the number, median latency, and median duration of SIs where the walking male or the non-walking male was the social partner.

#### Nonaggressive, social behaviors

To ensure effects specific to aggressive behaviors, we analyzed investigation-triggered SIs (**S5A–S5D Fig**). If recordings had more investigations than aggressive behaviors, we matched the number; if fewer, we used all investigations. We calculated the number, median latency, and median duration of SIs where the investigating male or the male being investigated was the social partner.

#### Random sampling procedure

To ensure that our results were not explained by large sample sizes or a subset of examples, we employed a permutation analysis (**S7A Fig**). We selected 50 sequences since the recording with the fewest aggression-triggered social interactions had 64 examples. Across all 11 recordings, we calculated the number of sequences where the aggressor and the aggressed males were the social partners within the subset. We then created an index value by taking the difference in the number of aggressive and aggressed sequences and dividing by the sum. This procedure was repeated 1,000 times to generate a distribution of index values and compared to a normal distribution (mean of 0, standard deviation of 1). We repeated this process for nonaggressive, nonsocial, and nonaggressive, social control analyses to ensure representative sampling (**S7B and S7C Fig**).

To ensure the state dependency of the results, we conducted a permutation analysis by randomizing aggressor identity while maintaining behavior order and duration. We calculated a difference index (difference in the number of aggressive and aggressed sequences divided by the sum) generated a distribution of index values through 1,000 permutations for each recording and compared to the observed difference index (**S7D Fig**).

#### Predicting behavioral state

We used decision tree classifiers to predict the behavioral state of the male social partner following triggering behaviors (aggressive encounters based on state or rank, walks, or investigations) (**Figs 2E and S4–S7E**). These calculations were performed using the Matlab fitctree function, with the duration of the social interaction and the latency between behavior and interaction as predictor variables. The outcome variable was the behavioral state of the male social partner. For each behavioral sequence type, we randomly selected 75% of the data as training data and 25% as testing data. Classifier accuracy was determined by dividing correct predictions by total predictions and converting to a percentage. This procedure was repeated 1,000 times to generate a distribution of accuracy values that was compared to chance levels. This procedure was applied to observed, sample-size-matched, and randomized data.

#### Predicting behavioral sequences

We used decision tree classifiers to predict the type of behavioral sequence (aggressive or control) (**S7D Fig**), applying the same procedure to observed, size-matched, and randomized data. Predictor variables included latency, duration, and the behavioral state of the male social partner, with the type of sequence as the outcome variable (chance level = 50%).

#### Temporal dynamics

To assess the temporal profile of behavioral sequences, we grouped examples into 5 one-hour bins (**Fig 5A**). We determined the proportion of events each hour where the aggressor or aggressed male was the social partner and assess differences using a Wilcoxon signed rank test. mcSVMs were trained using the Matlab fitcecoc function to decode the hour of occurrence using latency, duration, and male social partner identity as predictor variables (chance level = 20%) (**Fig 5B**). Within each hour, we also predicted the identity of the social partner using decision tree classifiers, trained on latency and duration of social interactions (chance level = 50%) (**Fig 5C**).

#### Three-step behavioral sequences

We characterized three-part behavioral sequences: an aggressive encounter, the social interaction between the aggressed male and a female, and the subsequent social interaction. Sequences were grouped into 4 types based on the participants in the second social interaction (**Table 2**). We measured the proportion (**Fig 6A**) and number (**Fig 6B**) of each sequence type across recordings.

To assess whether the observed proportions differed from chance (**Fig 6C**), we used temporally randomized data, generating a distribution of proportions for each shuffled iteration that was compared to the actual proportions.

We trained mcSVMs to decode sequence type using features of aggressive behavior (described in results, but not plotted), including average speed and total distance traveled by both the aggressor and aggressed. The outcome variable was sequence type (1–4), with chance-level accuracy at 25%. We used 75% of data for training and 25% for testing, repeating the procedure 1,000 times to generate a distribution of accuracy values that was compared to chance levels.

We also assessed inter-individual distance between both interacting males and females during the sequences (**Figs 7A–7B and S8A–S8F**). For type 1 sequences, we also measured the distance between the males (**Fig 7C and 7D**). Sequences were divided into 4 phases: aggressive encounter, latency between the aggressive encounter and the social interaction with the aggressed male, social interaction, and a five-second post-interaction period. Durations were normalized using the longest event in the phases. Distances were interpolated to match the normalized duration using Matlab’s interp1 function.

An mcSVM was trained to determine sequence type using inter-individual distances between aggressor/female and aggressed/female at the following time points: the start and end of the aggressive behavior, the start and end of the first social interaction, and the start and end of the second social interaction (**Fig 7E**). The outcome variable was sequence type (1–4), and chance-level accuracy was 25%. This procedure was performed 1,000 times to generate a distribution of accuracy values, which was compared to chance levels.

To evaluate the success of the behavioral strategy (**Fig 7F and 7G**), we calculated fight frequency after each sequence type. We compared the number and proportion of fights following type 1 sequences to other types (2, 3, and 4 combined because these sequences constituted 47% of the sequences).

#### Statistical comparisons

We used Kolmogorov–Smirnov tests and visual inspection to assess normality. For non-normal data, we applied nonparametric tests. Aggression levels between co-recorded males were evaluated using a Chi-square test. Paired data comparisons were conducted with a Wilcoxon signed rank test. To compare the results of permutation analyses and predictive models, we used a z-test with an alpha level of 0.05. For data requiring multiple comparisons, we employed a Kruskal–Wallis test with Dunn–Sidak post hoc correction.

## Supporting information

S1 Fig Mouse trajectories. Behavioral trajectories for each mouse across all recordings. Note, all mice explored the majority of the behavioral arena. Source data can be found in S1–S12 Datasets.
(DOCX)

S2 Fig Quantifying dynamic social relationships. (A) Aggression scores show which mouse acted as the aggressor in most aggressive encounters. Recordings sorted from smallest to largest score disparities. In recordings 3–11, highlighted with a gray box, one of the males was significantly more aggressive than the other. When comparing more and less aggressive animals, we only included recordings with significant differences (recordings 3–11). (B) Overall time spent in a submissive state. Black denotes the more aggressive male, while gray denotes the less aggressive male. (C) Number of aggressive behaviors per mouse. Lines connect co-recorded mice. The horizontal bars and boxes show the medians and interquartile ranges (25%–75%). Each data point represents the median of the distribution for each individual. Wilcoxon signed rank test: W = 45, *p* < 0.005. (D) Percentage of total aggression time co-recorded animals spent behaving as the aggressor. Wilcoxon signed rank test: W = 44, *p* < 0.01. (E) Left: Schematic of social interaction with females. Right: Number of social interactions (SI) between males and females. Wilcoxon signed rank test, W = 31, *p* = 0.36. (F) Time spent interacting with females. Wilcoxon signed rank test, W = 33, *p* = 0.25. (G) The median duration of social interactions for the more and less aggressive animals. Top: Median duration of social interactions for each animal. Bottom: Median across groups. Wilcoxon signed rank test, W = 34, *p* = 0.22. Numerical values for S2A–S2G Fig are available as an online supporting file (S1 Data). Source data can be found in S1–12 Datasets.
(DOCX)

S3 Fig Ethograms depict a dynamic social landscape. The top and bottom rows (red vertical lines, labeled F) show male and female interactions. Middle row denotes aggressive behaviors between males. Top of the central row (black lines, labeled A) shows acts of aggression from the more aggressive animals, while bottom of the central row (gray lines, labeled A) shows acts of aggression from the less aggressive animal. Interleaved rows (labeled S) indicate submissive states (blue patches). Steps in the submissive states indicate consecutive acts of aggression from the other male. Source data can be found in S1–12 Datasets.
(DOCX)

S4 Fig Nonaggressive, nonsocial triggers do not modulate subsequent interactions with females. (A) Schematic of nonaggressive, nonsocial sequences. Sequences consisted of a male walking in isolation followed by male–female social interactions. (B) The number of male–female interactions after walking or not walking. Lines connect co-recorded mice. Black lines and white boxes show the medians and interquartile ranges (25%–75%). Wilcoxon signed rank test, W = 47, *p* = 0.24. (C) The latency between walking or not walking behaviors and social interactions. Wilcoxon signed rank test, W = 21, *p* = 0.54. (D) The duration of social interactions following walking-triggered sequences. Wilcoxon signed rank test, W = 32, *p* = 0.68. (E) Performance of decoders when predicting the behavioral state of the male social partner in post-aggression social interactions. Black lines and white boxes show the means and standard deviations. The red line denotes chance levels. Each condition: 1-sided z-test, *n* = 1,000 iterations. Observed: z = 1.73, *p* = 0.04; size-matched: z = 1.11, *p* = 0.13; randomized: z = −0.31, *p* = 0.38. Numerical values for S4B–S4D Fig are available as an online supporting file (S1 Data). Source data can be found in S1–12 Datasets.
(DOCX)

S5 Fig Nonaggressive, social triggers do not modulate subsequent interactions with females. (A) Schematic of nonaggressive, social sequences. Sequences consisted of investigative male interactions followed by male–female social interactions. (B) The number of male–female interactions after investigating or being investigated. Lines connect co-recorded mice. Black lines and white boxes show the medians and interquartile ranges (25%–75%). Wilcoxon signed rank test, W = 39, *p* = 0.62. (C) The latency between investigating or being investigated and social interactions. Wilcoxon signed rank test, W = 51, *p* < 0.005. (D) The duration of social interactions following investigation-triggered sequences. Wilcoxon signed rank test, W = 54, *p* = 0.07. (E) Performance of decoders when predicting the behavioral state of the male social partner in post-aggression social interactions. Black lines and white boxes show the means and standard deviations. The red line denotes chance levels. Each condition: 1-sided z-test, *n* = 1,000 iterations. Observed: z = 0.47, *p* = 0.39; size-matched: z = 0.44, *p* = 0.33; randomized: z = −0.37, *p* = 0.36. Numerical values for S5B–S5D Fig are available as an online supporting file (S1 Data). Source data can be found in S1–12 Datasets.
(DOCX)

S6 Fig Aggregate aggression levels do not modulate subsequent interactions with females. (A) Schematic of aggressive social sequences. (B) The number of male–female interactions for the more or less aggressive male following aggressive behaviors. Lines connect co-recorded mice. The horizontal bars and boxes below the data show the medians and interquartile ranges (25%–75%). Wilcoxon signed rank test, W = 27, *p* = 0.30. (C) The latency between aggressive encounters and social interactions. Wilcoxon signed rank test, W = 31, *p* = 0.36. (D) The duration of social interactions following aggressive encounters. Wilcoxon signed rank test, W = 31, *p* = 0.36. (E) Decoders’ performance when predicting the aggregate aggression level of the male social partner in post-aggression social interactions. The horizontal bars and boxes below the data show the means and standard deviations. The red line denotes chance levels. Each condition: 1-sided z-test, *n* = 1,000 iterations. Observed: z = 0.21, *p* = 0.83; size-matched: z = −0.29, *p* = 0.77; randomized: z = 0.39, *p* = 0.70. Numerical values for S6B–S6D Fig are available as an online supporting file (S1 Data). Source data can be found in S1–12 Datasets.
(DOCX)

S7 Fig Aggressive-triggered sequences differ from walking- and investigation-triggered sequences. (A) A subsample of aggression-triggered sequences was randomly selected from each recording. Next, a difference index was calculated by subtracting the number of interactions between the aggressed male and female from the number of interactions between the aggressor male and female. The difference was then divided by the total to create an index. An index value below 0 reflects more interactions occurring between an aggressed male and female than an aggressor male and female. The random sampling procedure and subsequent index calculations were performed 1,000 times. Aggressed interactions with the female occurred significantly more often than aggressor interactions with the female, as the distribution was shifted to the left of zero. Two-sided z-test, *n* = 1,000 permutations, z = 7.85, *p* < 0.001. (B) As in A, for walking-triggered sequences. Indices were not significantly different from zero. z = −1.28, *p* = 0.21. (C) As in A, for investigation-triggered sequences. Indices were not significantly different from zero. z = 0.45, *p* = 0.65. (D) After randomly shuffling the identities of the aggressor and aggressed mice, we quantified the frequency that animals engaged in aggression-triggered social interactions and computed an index. This procedure was repeated 1,000 times. A z-score was calculated to compare the actual index value (denoted by a blue line) to the randomly generated distribution. Two-sided z-test, z-score = −11.05, *p* < 0.001. (E) Decoders’ performance when predicting aggressive or nonaggressive triggered sequences. The horizontal bars and boxes below the data show the means and standard deviations. The red line denotes chance levels. Each condition: 1-sided z-test, *n* = 1,000 iterations. Observed: z = 4.73, *p* < 0.001; size-matched: z = 4.32, *p* < 0.001; randomized: z = 0.05, *p* = 0.48. Source data can be found in S1–12 Datasets.
(DOCX)

S8 Fig Quantification of sequence types 2, 3, and 4. (A) For sequence type 2, distances between the aggressive or aggressed males and the female social partner were calculated during aggressive behaviors (AGG), the time between AGG and male-female social interactions, interactions, and 5 s post interaction. The lines and shaded regions show the medians and interquartile ranges (25%–75%). The dashed lines indicate times representing 1 s after the start of the social interaction and post-interaction times of 1, 2, and 3 s. (B) For sequence type 2, quantification of distances 1 s after the start of the social interaction and post-interaction times of 1, 2, and 3 s. Times correspond to the colored dashed lines in A. Top: all distances. Bottom: median distances for each mouse and compared using Wilcoxon signed rank test. start + 1 second: W = 29,521, *p* < 0.0001; end + 1 second: W = 29,028, *p* < 0.0001; end + 2 second: W = 28,036, *p* < 0.0001; end + 3 second: W = 26,450, *p* < 0.0001. (C) As in A, for sequence type 3. (D) As in B, for sequence type 3. start + 1 second: W = 6,210, *p* < 0.0001; end + 1 second: W = 5,980, *p* < 0.0001; end + 2 second: W = 5,702, *p* < 0.0001; end + 3 second: W = 4,995, *p* < 0.0001. (E) As in A, for sequence type 4. (F) As in B, for sequence type 4. start + 1 second: W = 19,306, *p* < 0.0001; end + 1 second: W = 16,730, *p* < 0.0001; end + 2 second: W = 5,358, *p* < 0.0001; end + 3 second: W = 14,307, *p* < 0.0001. Numerical values for S8B, S8D, and S8F Fig are available as an online supporting file (S1 Data). Source data can be found in S1–12 Datasets.
(DOCX)

S1 Table Comparing the number of aggressive behaviors between 2 males in a recording using a Chi-square test. Source data can be found in S1–12 Datasets.
(DOCX)

S1 Video Bait-and-switch sequence. Video shows a chase (aggressive, social behavior), a social interaction between the aggressed male and a female, and a subsequent interaction between the aggressor and the same female. The aggressor male’s fur is marked with 2 vertical stripes, while the aggressed is marked with 2 horizontal stripes. The female social partner is marked with a vertical slash. Video playback speed has been slowed to 15 frames per second.
(MP4)

S1 Code Compressed file with all the custom written Matlab scripts used to analyze the data. (ZIP)

S1 Data The numerical values used for each figure. (XLSX)

S1 Dataset Source data. (XLSX)

S2 Dataset Source data. (ZIP)

S3 Dataset Source data. (ZIP)

S4 Dataset Source data. (ZIP)

S5 Dataset Source data. (ZIP)

S6 Dataset Source data. (ZIP)

S7 Dataset Source data. (ZIP)

S8 Dataset Source data. (ZIP)

S9 Dataset Source data. (ZIP)

S10 Dataset Source data. (ZIP)

S11 Dataset Source data. (ZIP)

S12 Dataset Source data. (ZIP)
