# Supplementary material for: Mice employ a bait-and-switch escape mechanism to de-escalate social conflict
Source: PLoS Biol. 2024 Oct 15;22(10):e3002496. doi: 10.1371/journal.pbio.3002496 (PMC11479765; doi:10.1371/journal.pbio.3002496)
Supplement: S1 Fig — Behavioral trajectories for each mouse across all recordings. Note, all mice explored the majority of the behavioral arena. Source data can be found in S1–S12 Datasets. (DOCX) [file pbio.3002496.s001.docx]

**S1 Fig**

**
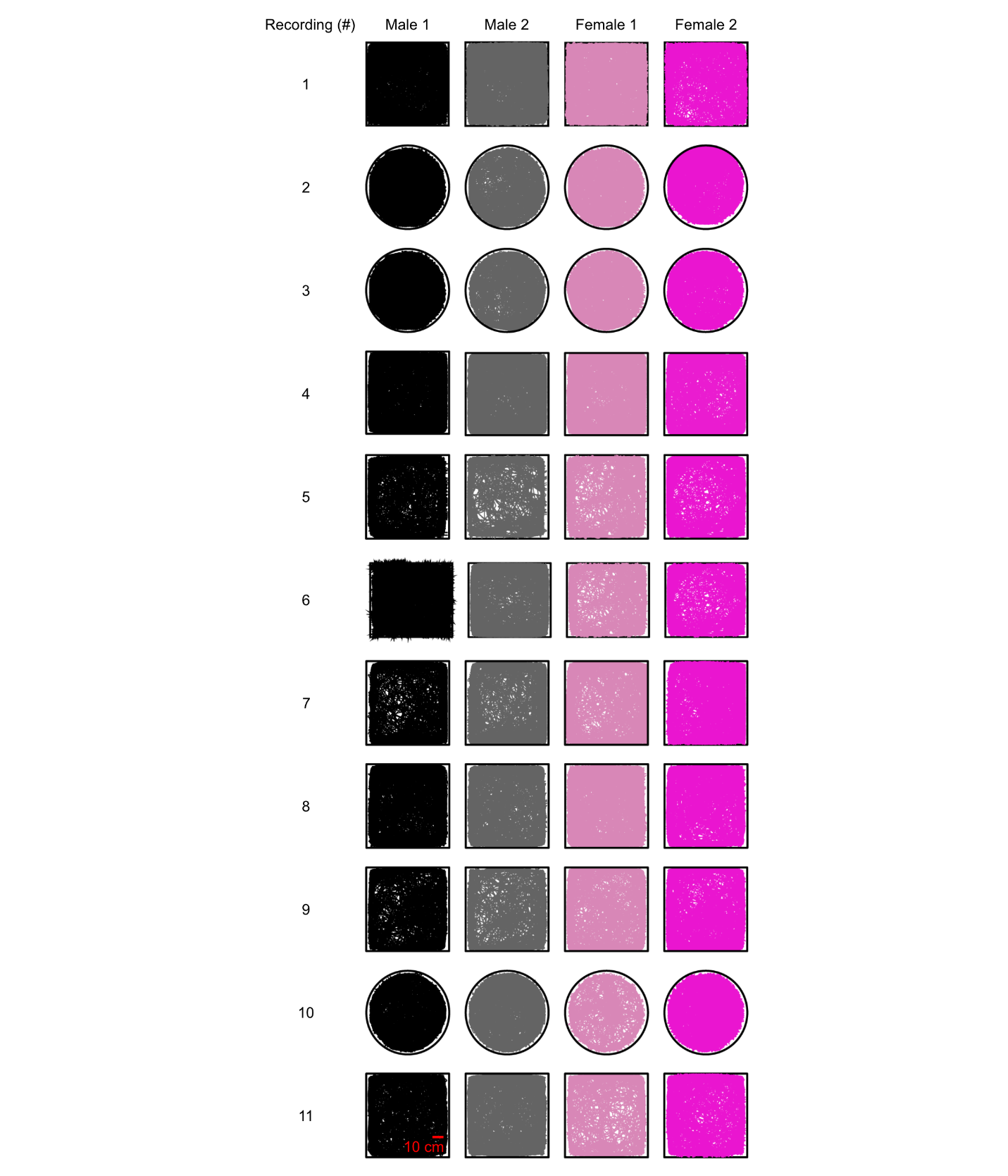
**

**S1 Fig. Mouse trajectories.**

Behavioral trajectories for each mouse across all recordings. Note, all mice explored the majority of the behavioral arena. Source data is available at S2_Data.zip.
