# Supplementary material for: Mice employ a bait-and-switch escape mechanism to de-escalate social conflict
Source: PLoS Biol. 2024 Oct 15;22(10):e3002496. doi: 10.1371/journal.pbio.3002496 (PMC11479765; doi:10.1371/journal.pbio.3002496)
Supplement: S2 Fig — (A) Aggression scores show which mouse acted as the aggressor in most aggressive encounters. Recordings sorted from smallest to largest score disparities. In recordings 3–11, highlighted with a gray box, one of the males was significantly more aggressive than the other. When comparing more and less aggressive animals, we only included recordings with significant differences (recordings 3–11). (B) Overall time spent in a submissive state. Black denotes the more aggressive male, while gray denotes the less aggressive male. (C) Number of aggressive behaviors per mouse. Lines connect co-recorded mice. The horizontal bars and boxes show the medians and interquartile ranges (25%–75%). Each data point represents the median of the distribution for each individual. Wilcoxon signed rank test: W = 45, p < 0.005. (D) Percentage of total aggression time co-recorded animals spent behaving as the aggressor. Wilcoxon signed rank test: W = 44, p < 0.01. (E) Left: Schematic of social interaction with females. Right: Number of social interactions (SI) between males and females. Wilcoxon signed rank test, W = 31, p = 0.36. (F) Time spent interacting with females. Wilcoxon signed rank test, W = 33, p = 0.25. (G) The median duration of social interactions for the more and less aggressive animals. Top: Median duration of social interactions for each animal. Bottom: Median across groups. Wilcoxon signed rank test, W = 34, p = 0.22. Numerical values for S2A–S2G Fig are available as an online supporting file (S1 Data). Source data can be found in S1–12 Datasets. (DOCX) [file pbio.3002496.s002.docx]

**S2 Fig**

**
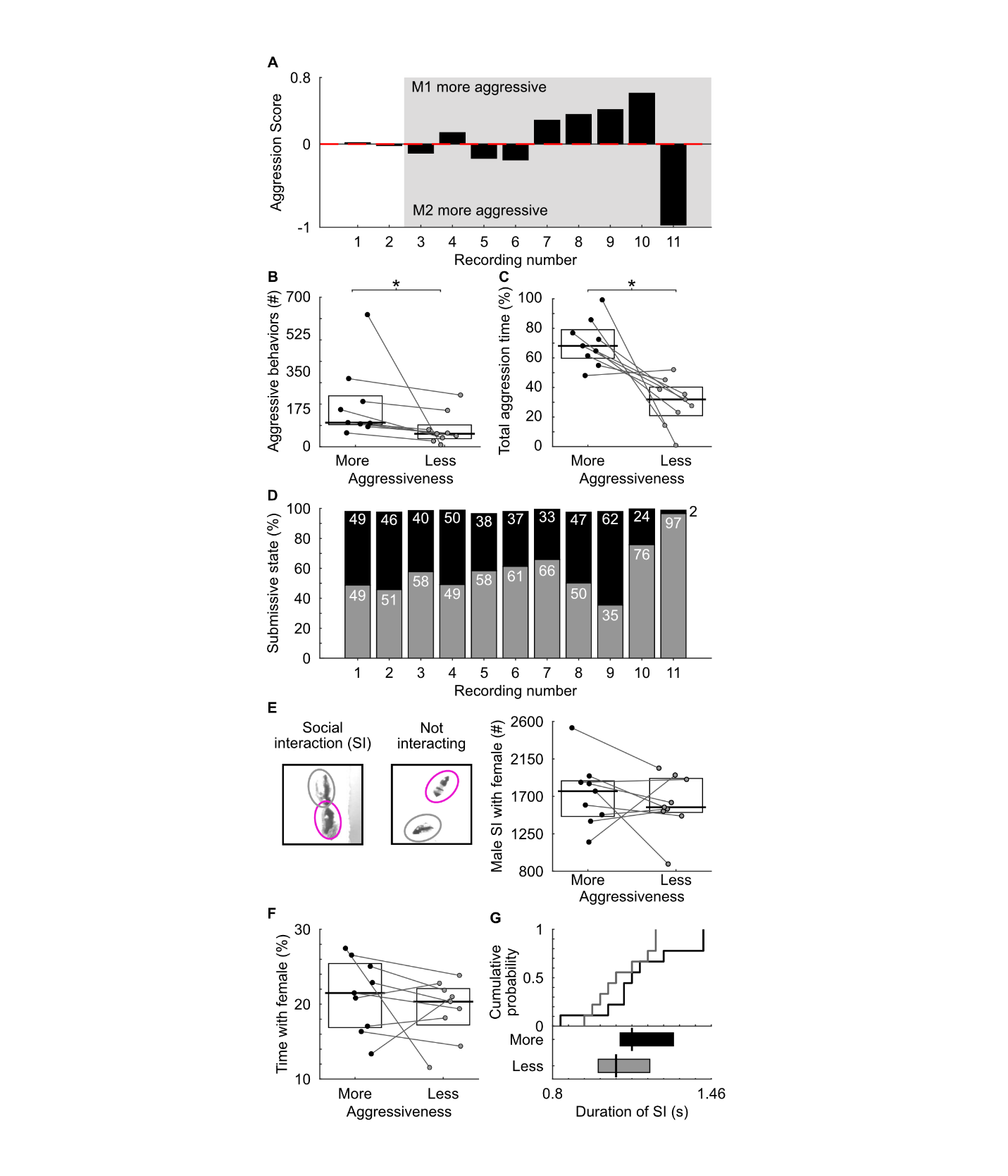
**

**S2 Fig. Quantifying dynamic social relationships.**

(A) Aggression scores show which mouse acted as the aggressor in most aggressive encounters. Recordings sorted from smallest to largest score disparities. In recordings 3-11, highlighted with a gray box, one of the males was significantly more aggressive than the other. When comparing more and less aggressive animals, we only included recordings with significant differences (recordings 3-11).

(B) Number of aggressive behaviors per mouse. Lines connect co-recorded mice. The horizontal bars and boxes show the medians and interquartile ranges (25-75%). Each data point represents the median of the distribution for each individual. Wilcoxon Signed Rank test: W = 45, p < 0.005

(C) Percentage of total aggression time co-recorded animals spent behaving as the aggressor. Wilcoxon Signed Rank test: W = 44, p < 0.01

(D) Overall time spent in a submissive state. Black denotes the more aggressive male, while gray denotes the less aggressive male.

(E) Left: Schematic of social interaction with females. Right: Number of social interactions (SI) between males and females. Wilcoxon Signed Rank test, W = 31, p = 0.36

(F) Time spent interacting with females. Wilcoxon Signed Rank test, W = 33, p = 0.25

(G) The median duration of social interactions for the more and less aggressive animals. Top: median duration of social interactions for each animal*.* Bottom: median across groups*.* Wilcoxon Signed Rank test, W = 34, p = 0.22

Numerical values for Figures S2A-S2G are available as an online supporting file (S1_Data.xlsx). Source data can be found in S2_Data.zip.
