# Supplementary material for: Mice employ a bait-and-switch escape mechanism to de-escalate social conflict
Source: PLoS Biol. 2024 Oct 15;22(10):e3002496. doi: 10.1371/journal.pbio.3002496 (PMC11479765; doi:10.1371/journal.pbio.3002496)
Supplement: S3 Fig — The top and bottom rows (red vertical lines, labeled F) show male and female interactions. Middle row denotes aggressive behaviors between males. Top of the central row (black lines, labeled A) shows acts of aggression from the more aggressive animals, while bottom of the central row (gray lines, labeled A) shows acts of aggression from the less aggressive animal. Interleaved rows (labeled S) indicate submissive states (blue patches). Steps in the submissive states indicate consecutive acts of aggression from the other male. Source data can be found in S1–12 Datasets. (DOCX) [file pbio.3002496.s003.docx]

**S3 Fig**


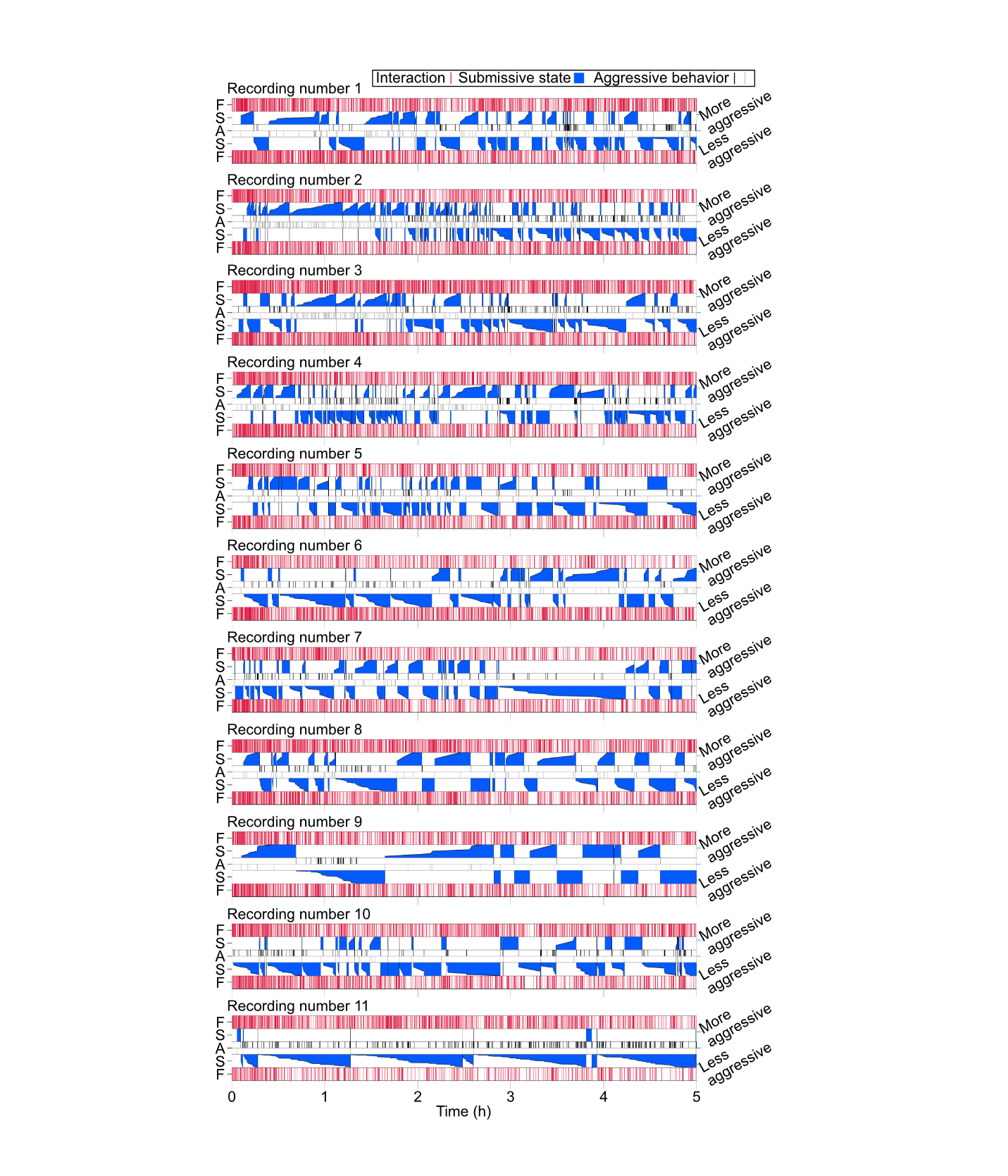


**S3 Fig. Ethograms depict a dynamic social landscape.**

The top and bottom rows (red vertical lines, labeled F) show male and female interactions. Middle row denotes aggressive behaviors between males. Top of the central row (black lines, labeled A) shows acts of aggression from the more aggressive animals, while bottom of the central row (gray lines, labeled A) shows acts of aggression from the less aggressive animal. Interleaved rows (labeled S) indicate submissive states (blue patches). Steps in the submissive states indicate consecutive acts of aggression from the other male. Source data can be found in S2_Data.zip.
