# Supplementary material for: Mice employ a bait-and-switch escape mechanism to de-escalate social conflict
Source: PLoS Biol. 2024 Oct 15;22(10):e3002496. doi: 10.1371/journal.pbio.3002496 (PMC11479765; doi:10.1371/journal.pbio.3002496)
Supplement: S4 Fig — (A) Schematic of nonaggressive, nonsocial sequences. Sequences consisted of a male walking in isolation followed by male–female social interactions. (B) The number of male–female interactions after walking or not walking. Lines connect co-recorded mice. Black lines and white boxes show the medians and interquartile ranges (25%–75%). Wilcoxon signed rank test, W = 47, p = 0.24. (C) The latency between walking or not walking behaviors and social interactions. Wilcoxon signed rank test, W = 21, p = 0.54. (D) The duration of social interactions following walking-triggered sequences. Wilcoxon signed rank test, W = 32, p = 0.68. (E) Performance of decoders when predicting the behavioral state of the male social partner in post-aggression social interactions. Black lines and white boxes show the means and standard deviations. The red line denotes chance levels. Each condition: 1-sided z-test, n = 1,000 iterations. Observed: z = 1.73, p = 0.04; size-matched: z = 1.11, p = 0.13; randomized: z = −0.31, p = 0.38. Numerical values for S4B–S4D Fig are available as an online supporting file (S1 Data). Source data can be found in S1–12 Datasets. (DOCX) [file pbio.3002496.s004.docx]

**S4 Fig**


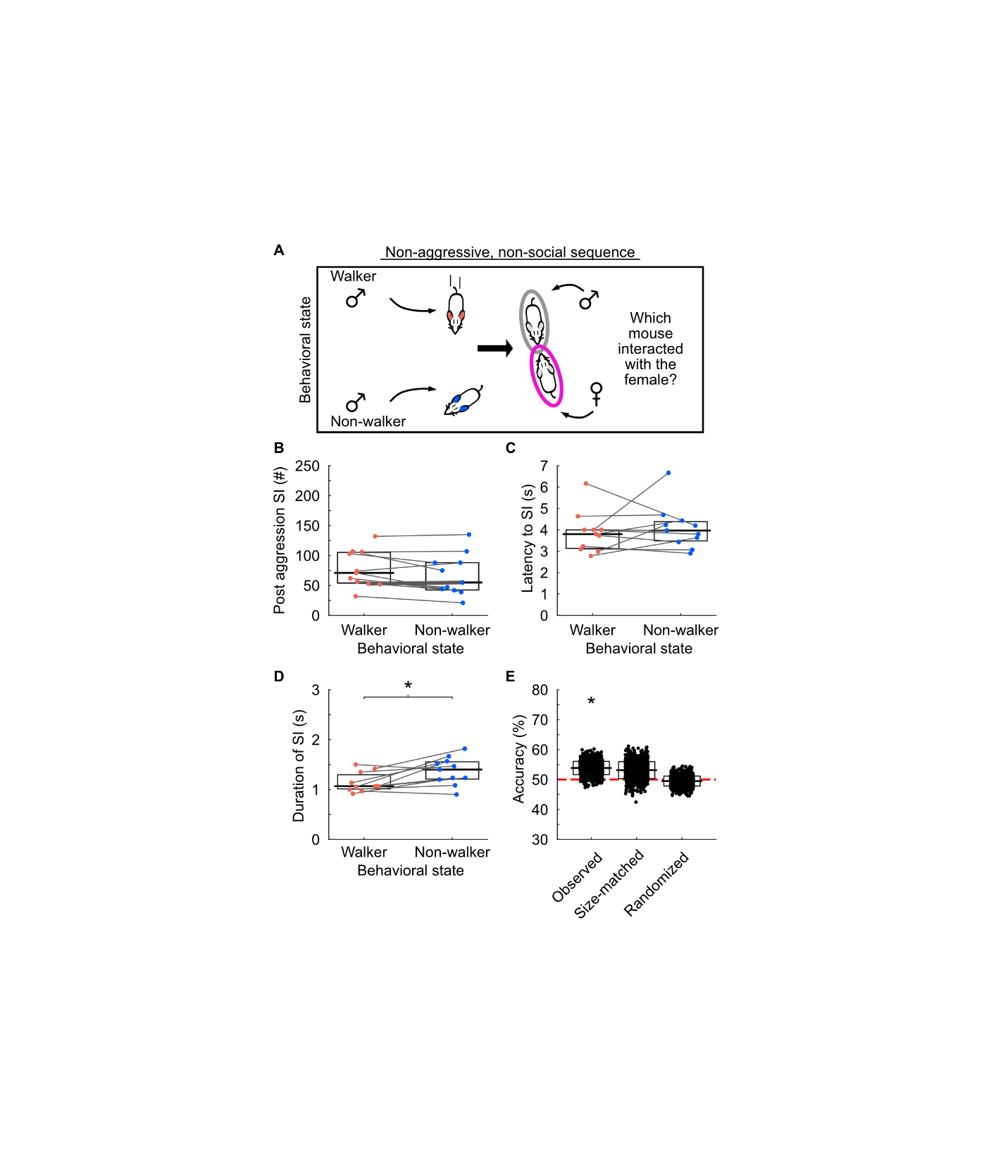


**S4 Fig. Non-aggressive, non-social triggers do not modulate subsequent interactions with females.**

(A) Schematic of non-aggressive, non-social sequences. Sequences consisted of a male walking in isolation followed by male-female social interactions.

(B) The number of male-female interactions after walking or not walking. Lines connect co-recorded mice. Black lines and white boxes show the medians and interquartile ranges (25-75%). Wilcoxon Signed Rank test, W = 47, p = 0.24

(C) The latency between walking or not walking behaviors and social interactions. Wilcoxon Signed Rank test, W = 21, p = 0.54

(D) The duration of social interactions following walking-triggered sequences. Wilcoxon Signed Rank test, W = 32, p = 0.68

(E) Performance of decoders when predicting the behavioral state of the male social partner in post-aggression social interactions. Black lines and white boxes show the means and standard deviations. The red line denotes chance levels. Each condition: 1-sided z-test, n = 1,000 iterations.

observed: z = 1.73, p = 0.04

size-matched: z = 1.11, p = 0.13

randomized: z = -0.31, p = 0.38

Numerical values for Figures S4B-S4D are available as an online supporting file (S1_Data.xlsx). Source data can be found in S2_Data.zip.
