# Supplementary material for: Mice employ a bait-and-switch escape mechanism to de-escalate social conflict
Source: PLoS Biol. 2024 Oct 15;22(10):e3002496. doi: 10.1371/journal.pbio.3002496 (PMC11479765; doi:10.1371/journal.pbio.3002496)
Supplement: S5 Fig — (A) Schematic of nonaggressive, social sequences. Sequences consisted of investigative male interactions followed by male–female social interactions. (B) The number of male–female interactions after investigating or being investigated. Lines connect co-recorded mice. Black lines and white boxes show the medians and interquartile ranges (25%–75%). Wilcoxon signed rank test, W = 39, p = 0.62. (C) The latency between investigating or being investigated and social interactions. Wilcoxon signed rank test, W = 51, p < 0.005. (D) The duration of social interactions following investigation-triggered sequences. Wilcoxon signed rank test, W = 54, p = 0.07. (E) Performance of decoders when predicting the behavioral state of the male social partner in post-aggression social interactions. Black lines and white boxes show the means and standard deviations. The red line denotes chance levels. Each condition: 1-sided z-test, n = 1,000 iterations. Observed: z = 0.47, p = 0.39; size-matched: z = 0.44, p = 0.33; randomized: z = −0.37, p = 0.36. Numerical values for S5B–S5D Fig are available as an online supporting file (S1 Data). Source data can be found in S1–12 Datasets. (DOCX) [file pbio.3002496.s005.docx]

**S5 Fig**

**
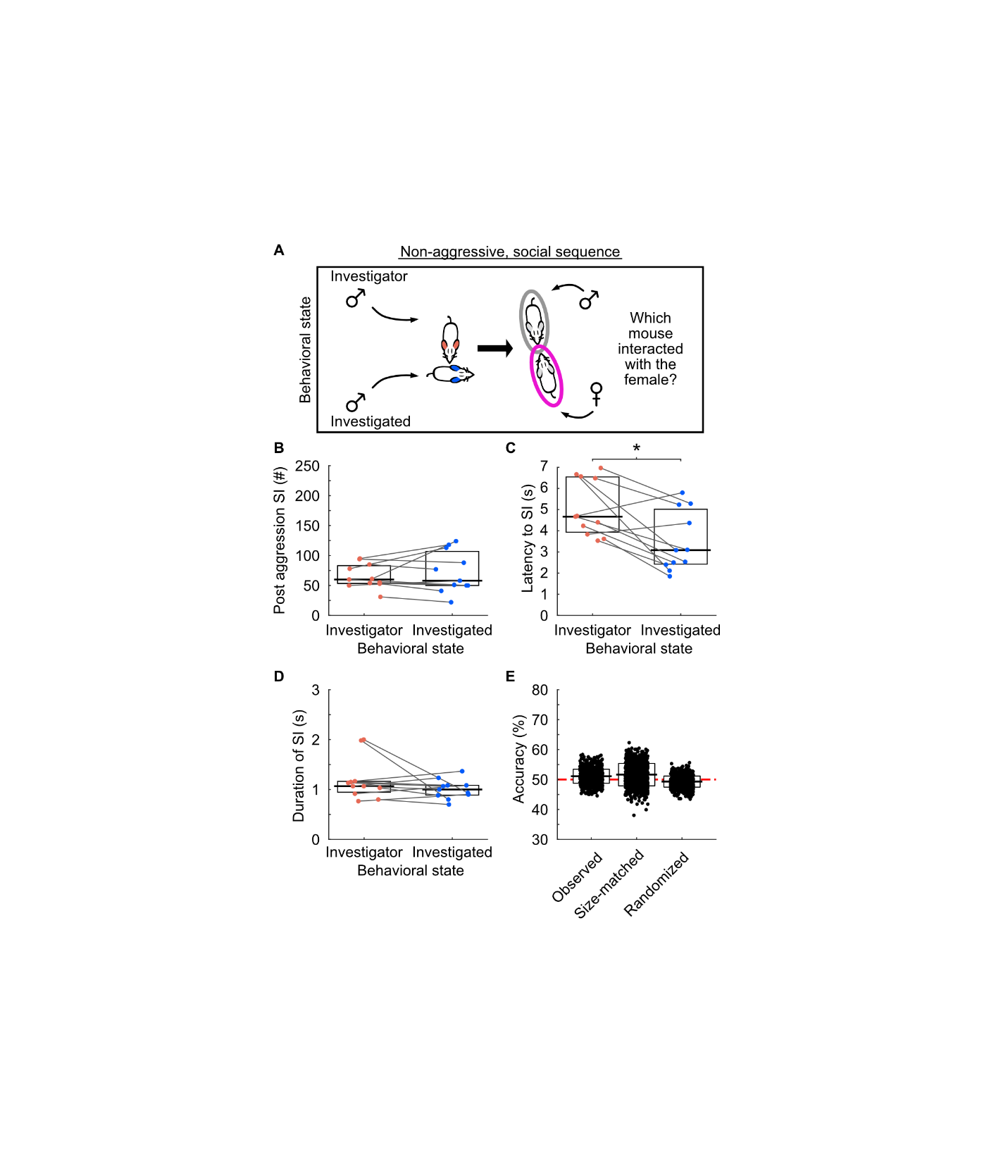
**

**S5 Fig. Non-aggressive, social triggers do not modulate subsequent interactions with females.**

(A) Schematic of non-aggressive, social sequences. Sequences consisted of investigative male interactions followed by male-female social interactions.

(B) The number of male-female interactions after investigating or being investigated. Lines connect co-recorded mice. Black lines and white boxes show the medians and interquartile ranges (25-75%). Wilcoxon Signed Rank test, W = 39, p = 0.62

(C) The latency between investigating or being investigated and social interactions. Wilcoxon Signed Rank test, W = 51, p < 0.005

(D) The duration of social interactions following investigation-triggered sequences. Wilcoxon Signed Rank test, W = 54, p = 0.07

(E) Performance of decoders when predicting the behavioral state of the male social partner in post-aggression social interactions. Black lines and white boxes show the means and standard deviations. The red line denotes chance levels. Each condition: 1-sided z-test, n = 1,000 iterations.

observed: z = 0.47, p = 0.39

size-matched: z = 0.44, p = 0.33

randomized: z = -0.37, p = 0.36

Numerical values for Figures S5B-S5D are available as an online supporting file (S1_Data.xlsx). Source data can be found in S2_Data.zip.
