# Supplementary material for: Mice employ a bait-and-switch escape mechanism to de-escalate social conflict
Source: PLoS Biol. 2024 Oct 15;22(10):e3002496. doi: 10.1371/journal.pbio.3002496 (PMC11479765; doi:10.1371/journal.pbio.3002496)
Supplement: S6 Fig — (A) Schematic of aggressive social sequences. (B) The number of male–female interactions for the more or less aggressive male following aggressive behaviors. Lines connect co-recorded mice. The horizontal bars and boxes below the data show the medians and interquartile ranges (25%–75%). Wilcoxon signed rank test, W = 27, p = 0.30. (C) The latency between aggressive encounters and social interactions. Wilcoxon signed rank test, W = 31, p = 0.36. (D) The duration of social interactions following aggressive encounters. Wilcoxon signed rank test, W = 31, p = 0.36. (E) Decoders’ performance when predicting the aggregate aggression level of the male social partner in post-aggression social interactions. The horizontal bars and boxes below the data show the means and standard deviations. The red line denotes chance levels. Each condition: 1-sided z-test, n = 1,000 iterations. Observed: z = 0.21, p = 0.83; size-matched: z = −0.29, p = 0.77; randomized: z = 0.39, p = 0.70. Numerical values for S6B–S6D Fig are available as an online supporting file (S1 Data). Source data can be found in S1–12 Datasets. (DOCX) [file pbio.3002496.s006.docx]

**S6 Fig**


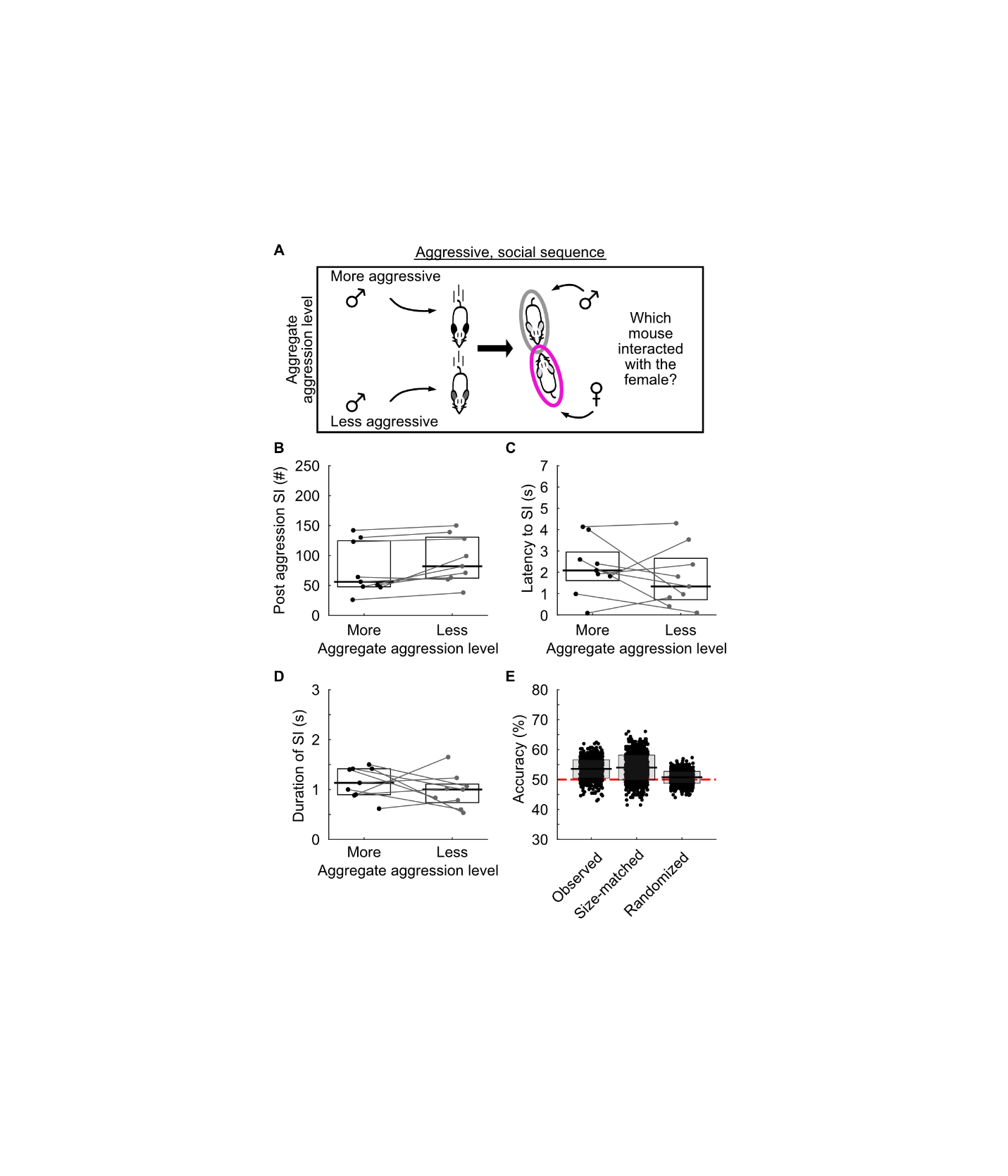


**S6 Fig. Aggregate aggression levels do not modulate subsequent interactions with females.**

(A) Schematic of aggressive social sequences.

(B) The number of male-female interactions for the more or less aggressive male following aggressive behaviors. Lines connect co-recorded mice. The horizontal bars and boxes below the data show the medians and interquartile ranges (25-75%). Wilcoxon Signed Rank test, W = 27, p = 0.30

(C) The latency between aggressive encounters and social interactions. Wilcoxon Signed Rank test, W = 31, p = 0.36

(D) The duration of social interactions following aggressive encounters. Wilcoxon Signed Rank test, W = 31, p = 0.36

(E) Decoders' performance when predicting the aggregate aggression level of the male social partner in post-aggression social interactions. The horizontal bars and boxes below the data show the means and standard deviations. The red line denotes chance levels. Each condition: 1-sided z-test, n = 1,000 iterations.

observed: z = 0.21, p = 0.83

size-matched: z = -0.29, p = 0.77

randomized: z = 0.39, p = 0.70

Numerical values for Figures S6B-S5D are available as an online supporting file (S1_Data.xlsx). Source data can be found in S2_Data.zip.
