# Supplementary material for: Mice employ a bait-and-switch escape mechanism to de-escalate social conflict
Source: PLoS Biol. 2024 Oct 15;22(10):e3002496. doi: 10.1371/journal.pbio.3002496 (PMC11479765; doi:10.1371/journal.pbio.3002496)
Supplement: S7 Fig — (A) A subsample of aggression-triggered sequences was randomly selected from each recording. Next, a difference index was calculated by subtracting the number of interactions between the aggressed male and female from the number of interactions between the aggressor male and female. The difference was then divided by the total to create an index. An index value below 0 reflects more interactions occurring between an aggressed male and female than an aggressor male and female. The random sampling procedure and subsequent index calculations were performed 1,000 times. Aggressed interactions with the female occurred significantly more often than aggressor interactions with the female, as the distribution was shifted to the left of zero. Two-sided z-test, n = 1,000 permutations, z = 7.85, p < 0.001. (B) As in A, for walking-triggered sequences. Indices were not significantly different from zero. z = −1.28, p = 0.21. (C) As in A, for investigation-triggered sequences. Indices were not significantly different from zero. z = 0.45, p = 0.65. (D) After randomly shuffling the identities of the aggressor and aggressed mice, we quantified the frequency that animals engaged in aggression-triggered social interactions and computed an index. This procedure was repeated 1,000 times. A z-score was calculated to compare the actual index value (denoted by a blue line) to the randomly generated distribution. Two-sided z-test, z-score = −11.05, p < 0.001. (E) Decoders’ performance when predicting aggressive or nonaggressive triggered sequences. The horizontal bars and boxes below the data show the means and standard deviations. The red line denotes chance levels. Each condition: 1-sided z-test, n = 1,000 iterations. Observed: z = 4.73, p < 0.001; size-matched: z = 4.32, p < 0.001; randomized: z = 0.05, p = 0.48. Source data can be found in S1–12 Datasets. (DOCX) [file pbio.3002496.s007.docx]

**S7 Fig**

**
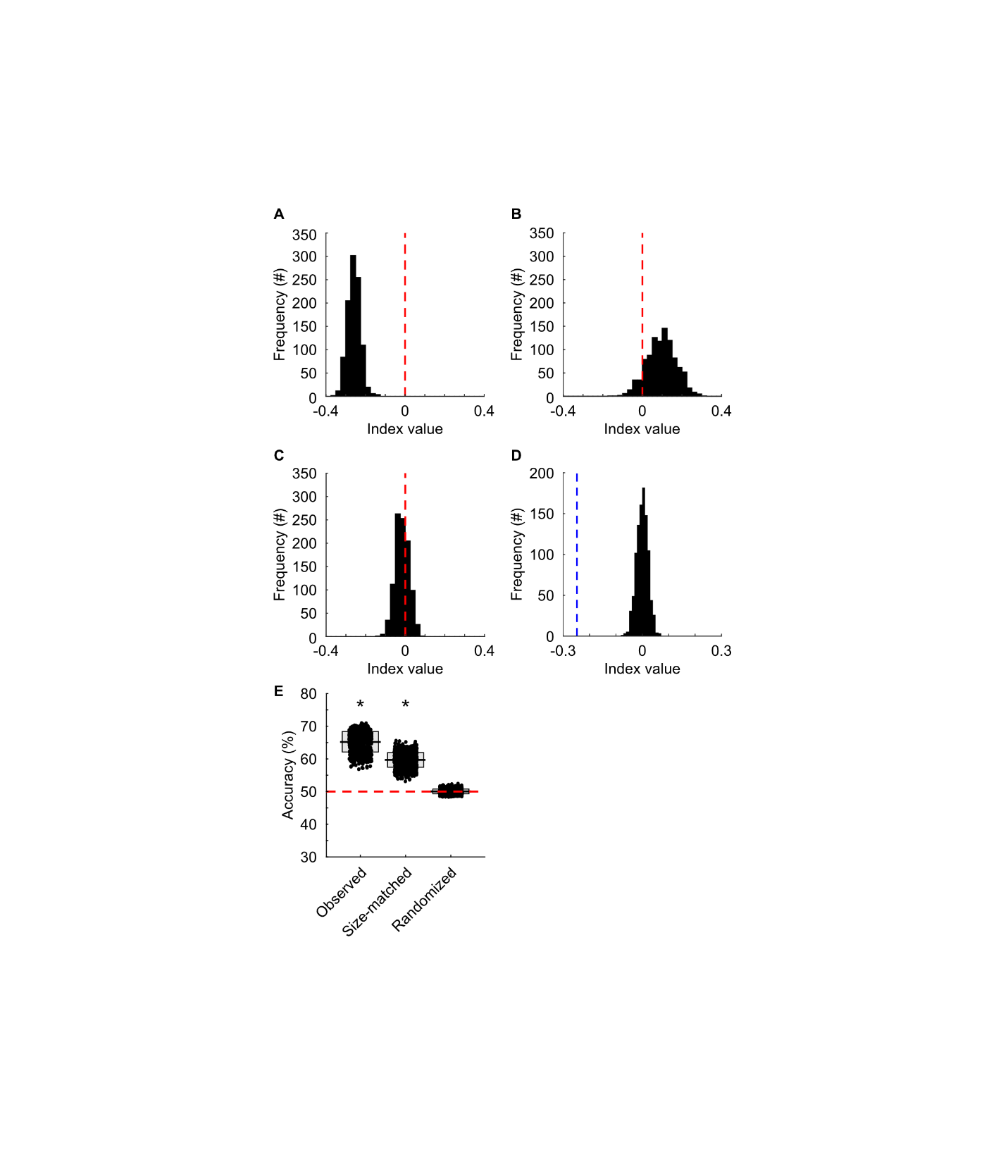
**

**S7 Fig. Aggressive-triggered sequences differ from walking- and investigation-triggered sequences.**

(A) A subsample of aggression-triggered sequences was randomly selected from each recording. Next, a difference index was calculated by subtracting the number of interactions between the aggressed male and female from the number of interactions between the aggressor male and female. The difference was then divided by the total to create an index. An index value below 0 reflects more interactions occurring between an aggressed male and female than an aggressor male and female. The random sampling procedure and subsequent index calculations were performed 1,000 times. Aggressed interactions with the female occurred significantly more often than aggressor interactions with the female, as the distribution was shifted to the left of zero. 2-sided z-test, n = 1,000 permutations, z = 7.85, p < 0.001

(B) As in A, for walking-triggered sequences. Indices were not significantly different from zero. z = -1.28, p = 0.21

(C) As in A, for investigation-triggered sequences. Indices were not significantly different from zero. z = 0.45, p = 0.65

(D) After randomly shuffling the identities of the aggressor and aggressed mice, we quantified the frequency that animals engaged in aggression-triggered social interactions and computed an index. This procedure was repeated 1,000 times. A z-score was calculated to compare the actual index value (denoted by a blue line) to the randomly generated distribution. 2-sided z-test, z-score = -11.05, p < 0.001

(E) Decoders' performance when predicting aggressive or non-aggressive triggered sequences. The horizontal bars and boxes below the data show the means and standard deviations. The red line denotes chance levels. Each condition: 1-sided z-test, n = 1,000 iterations.

observed: z = 4.73, p < 0.001

size-matched: z = 4.32, p < 0.001

randomized: z = 0.05, p = 0.48

Source data can be found in S2_Data.zip.
