# Supplementary material for: Mice employ a bait-and-switch escape mechanism to de-escalate social conflict
Source: PLoS Biol. 2024 Oct 15;22(10):e3002496. doi: 10.1371/journal.pbio.3002496 (PMC11479765; doi:10.1371/journal.pbio.3002496)
Supplement: S8 Fig — (A) For sequence type 2, distances between the aggressive or aggressed males and the female social partner were calculated during aggressive behaviors (AGG), the time between AGG and male-female social interactions, interactions, and 5 s post interaction. The lines and shaded regions show the medians and interquartile ranges (25%–75%). The dashed lines indicate times representing 1 s after the start of the social interaction and post-interaction times of 1, 2, and 3 s. (B) For sequence type 2, quantification of distances 1 s after the start of the social interaction and post-interaction times of 1, 2, and 3 s. Times correspond to the colored dashed lines in A. Top: all distances. Bottom: median distances for each mouse and compared using Wilcoxon signed rank test. start + 1 second: W = 29,521, p < 0.0001; end + 1 second: W = 29,028, p < 0.0001; end + 2 second: W = 28,036, p < 0.0001; end + 3 second: W = 26,450, p < 0.0001. (C) As in A, for sequence type 3. (D) As in B, for sequence type 3. start + 1 second: W = 6,210, p < 0.0001; end + 1 second: W = 5,980, p < 0.0001; end + 2 second: W = 5,702, p < 0.0001; end + 3 second: W = 4,995, p < 0.0001. (E) As in A, for sequence type 4. (F) As in B, for sequence type 4. start + 1 second: W = 19,306, p < 0.0001; end + 1 second: W = 16,730, p < 0.0001; end + 2 second: W = 5,358, p < 0.0001; end + 3 second: W = 14,307, p < 0.0001. Numerical values for S8B, S8D, and S8F Fig are available as an online supporting file (S1 Data). Source data can be found in S1–12 Datasets. (DOCX) [file pbio.3002496.s008.docx]

**S8 Fig**


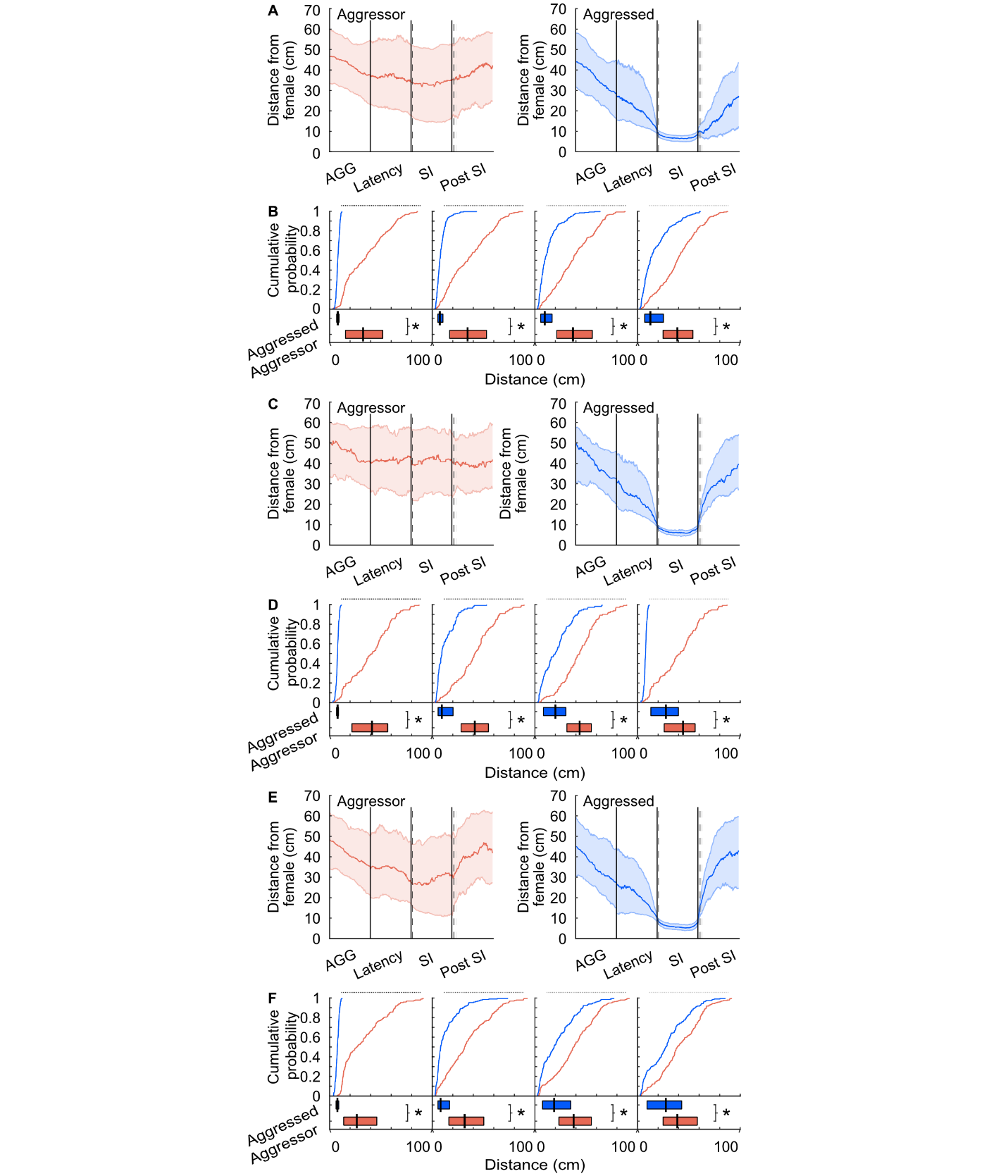


**S8 Fig. Quantification of sequence types 2, 3, and 4.**

(A) For sequence type 2, distances between the aggressive or aggressed males and the female social partner were calculated during aggressive behaviors (AGG), the time between AGG and male-female social interactions, interactions, and 5 seconds post interaction. The lines and shaded regions show the medians and interquartile ranges (25-75%). The dashed lines indicate times representing one second after the start of the social interaction and post-interaction times of 1, 2, and 3 seconds.

(B) For sequence type 2, quantification of distances one second after the start of the social interaction and post-interaction times of 1, 2, and 3 seconds. Times correspond to the colored dashed lines in A. Top: all distances. Bottom: median distances for each mouse and compared using Wilcoxon Signed Rank test.

start + 1 second: W = 29,521, p < 0.0001

end + 1 second: W = 29,028, p < 0.0001

end + 2 second: W = 28,036, p < 0.0001

end + 3 second: W = 26,450, p < 0.0001

(C) As in A, for sequence type 3.

(D) As in B, for sequence type 3.

start + 1 second: W = 6,210, p < 0.0001

end + 1 second: W = 5,980, p < 0.0001

end + 2 second: W = 5,702, p < 0.0001

end + 3 second: W = 4,995, p < 0.0001

(E) As in A, for sequence type 4.

(F) As in B, for sequence type 4.

start + 1 second: W = 19,306, p < 0.0001

end + 1 second: W = 16,730, p < 0.0001

end + 2 second: W = 5,358, p < 0.0001

end + 3 second: W = 14,307, p < 0.0001

Numerical values for Figures S8B, S8D, and S8F are available as an online supporting file (S1_Data.xlsx). Source data can be found in S2_Data.zip.
