# Supplementary material for: Mice employ a bait-and-switch escape mechanism to de-escalate social conflict
Source: PLoS Biol. 2024 Oct 15;22(10):e3002496. doi: 10.1371/journal.pbio.3002496 (PMC11479765; doi:10.1371/journal.pbio.3002496)
Supplement: S1 Table — Source data can be found in S1–12 Datasets. (DOCX) [file pbio.3002496.s009.docx]

**S1 Table. Comparing the number of aggressive behaviors between two males in a recording using a Chi-Square test.**

|  | Observed | | Expected | |  |  |
| --- | --- | --- | --- | --- | --- | --- |
| Recording | Male 1 | Male 2 | Male 1 | Male 2 | Statistic | P |
| 1 | 165 | 160 | 162.5 | 162.5 | 0.08 | 0.78 |
| 2 | 260 | 270 | 265 | 265 | 0.19 | 0.66 |
| 3 | 170 | 212 | 191 | 191 | 4.62 | 0.031 |
| 4 | 319 | 242 | 280.5 | 280.5 | 10.57 | 0.0012 |
| 5 | 80 | 113 | 96.5 | 96.5 | 5.64 | 0.018 |
| 6 | 64 | 94 | 79 | 79 | 5.70 | 0.017 |
| 7 | 110 | 61 | 85.5 | 85.5 | 14.04 | 0.00018 |
| 8 | 107 | 51 | 79 | 79 | 19.85 | 0.0000084 |
| 9 | 65 | 27 | 46 | 46 | 15.70 | 0.000074 |
| 10 | 174 | 42 | 108 | 108 | 80.67 | <0.000001 |
| 11 | 9 | 618 | 313.5 | 313.5 | 591.52 | <0.000001 |
