# Supplementary material for: Mice employ a bait-and-switch escape mechanism to de-escalate social conflict
Source: PLoS Biol. 2024 Oct 15;22(10):e3002496. doi: 10.1371/journal.pbio.3002496 (PMC11479765; doi:10.1371/journal.pbio.3002496)
Supplement: S1 Code — (ZIP) [file pbio.3002496.s011.zip › S1_Code/README.pdf]

Figure 1

script_occupancycode – occupancy analysis presented in Figure 1D; calculates the percentage of the arena explored by each animal by dividing the arena into equally spaced bins and determining how many bins were entered.

Figure 2

script_forAGGsequence – calculates sequences where there is an aggressive behavior followed by a social interaction and determines the actors, latency between behaviors, and duration of the social interaction. Also makes various plots.

script_runSVMclassifier_sequences_figure2 – runs a series of SVM classifiers to predict the behavioral state of the male social partner in the social interaction of a sequence. Can load in pre-existing structures to run this on any type of sequence (aggression, walk, or investigation triggered). Lines 74 and 75 can be modified if you wish to use status rather than state.

Figure 3

Script_calculateangle – calculates difference in theta to determine whether mice are facing the same direction at a specific point in time

Script_mfangle – determines the size of the angle between the male and the female by creating a vector. Values are computed for aggressor and aggressed males at a specific time point. Requires the fn_determineangle function, which also calls find_nose_tail_jpn. Heading direction.

Figure 4

Script_determineinitiatorproportion – calculates proportion of interactions initiated by males or females. Requires structure detailing initiator identities for all social interactions (neunuebel lab specific – available upon request)

Script_femaleproportionofevents – calculates number of times the aggressor or aggressed interacted with either female 1 or female, and then determines the relative proportion of interactions between aggressor/aggressed and either female.

Script_latencytozone – determine latency between zone entrance and end of aggressive behavior for aggressors and aggressed after calling the structure created by script_getoverlapwithzone. Requires a list of aggressive encounters.

Figure 5

Script_numberofsequences_overtime – determines the proportion of times aggressor/aggressed (or walker/non-walker/investigator/investigated) engaged in a sequence within each time bin, relative to the total number of sequences in that bin. Loads in a pre-made structure that has the start and end time of trigger behaviors and social interactions. Also contains code to determine whether the latency/duration changes across bins. Bin size is a flexible variable. Also can be used for supplemental figure 4.

script_classifierfigures_fortimebin – makes beeswarm figures

script_classifiercode_temporalproportions_predictwithintimebins – uses features of the sequence to predict the time bin in which they began. Flexible for different sequence types, and runs a subsample and shuffle control.

Figure 6

Script_behavioroutcomeshuffle – determines the number/proportion of each sequence type with the option to shuffle the types. Sequences types are calculated at **script_outcomes_postBS.**

Figure 7

Script_summaryplots_trajectoriessequences – calculates distance between males and females at particular points in time. Requires input of a structure with sequence data. Set time points/type of sequence as variables. Requires fn_distancefromfemale function and other prep functions. Also used in supplemental figure 8.

Script_trajectory_sequence1 – interpolates the length of all sequences to match the length of the longest sequence across all recordings, and then finds the distance between animals throughout the sequence. Also used in supplemental figure 8.

Script_mmdistanceplots – calculates distance between males at particular points in time. Requires input of a structure with sequence data. Requires fn_updatedinterpolation_withmm and other prep functions.

Script_mm_distanceplot_comparisons – compares distances between males at different time points and makes CDF/IQR plots.

Script_runsvmclassifier_behavioroutcomes – calculates distances between animals at specific points in time and then uses these values as input to an SVM tasked with predicting the type of sequence. The script runs the classifier 1000 times, on either all of the data, a subsample of the data, or data in which the sequence type has been randomized.

Script_baitandswitch_fight – finds fights that occur around the onset of a sequence and computes comparisons between sequence types

Supplemental Figure 2

script_determinestates – makes a list of the aggressive and submissive states throughout the recording, and calculates the duration of time each mouse spent behaving aggressively. Also determines the number, total duration, and median duration of male-female interactions for both the overall dominant and overall submissive male. Includes code to generate figures.

script_AGGindex – calculates aggression index for each experiment based on the number of behaviors, also reports the number of aggressive behaviors performed by each mouse.

Supplemental Figure 4

script_walk_control – calculates sequences using walks in the place of aggressive behavior. Randomly selects a number of walks that matches the number of aggressive behaviors in a given experiment.

Supplemental Figure 5

script_socialcontrol_MIM – calculates sequences using male investigate male behaviors in the place of aggressive encounters.

Supplemental Figure 6

Script_AGGscript_domsub - calculates sequences where there is an aggressive behavior followed by a social interaction and determines the actors based on aggregate aggression levels, latency between behaviors, and duration of the social interaction. Also makes various plots.

Supplemental Figure 7

script_samplesizecontrol_aggdata – sample size control script; gets a random number of behaviors from each recording (based on recording with smallest amount of sequences), and calculates an index value. Also does this by shuffling the data. Can be modified to work for walks or investigations.

script_indexvalue_shuffleidentity – code for calculating an index value for the total number of aggressor/aggressed sequences. also contains code to shuffle the identity of the aggressor and re-calculate the index value. This procedure is performed 1000 times to create a distribution of values. Can be modified to work for investigates or walks.

script_predictsequence – code for predicting whether a sequence was aggression triggered or non-aggression triggered (walk/investigation) by using the latency between the trigger behavior and the social interaction, and the duration of the social interaction. Loads in structures located on the Z drive, and curates the data, and then runs a decision tree classifier on all of the data, a subsample of the data (where the number of aggression-triggered sequences and non-aggression triggered sequences are sample-matched), and data where the identity of the aggressor/aggressor proxy was randomized.

Methodology

SocialEllipseProgram-GroupSI – folder that contains code for extracting social interactions and assigning initiators. Necessary functions are included in the toolbox. Run MouseLocGrp.

script_extract_flee_from_JAABA_output – extracts flee events and creates a spreadsheet

script_extract_walk_from_JAABA_output – extracts walk events and creates a spreadsheet

script_extract_male_male_chases_from_JAABA_output – extracts chase events and creates a spreadsheet

script_extract_fights_from_JAABA_output – extracts fight events and creates a spreadsheet

script_extract_male_investigate_from_JAABA_output – extracts investigation events and creates a spreadsheet

*Toolbox functions:

- calc_traj_ovlp – function necessary for loading tracking data and getting distances

- closest_mouse - function necessary for social interaction program; calculates closest mouse

- deriv – necessary for loading track function

- fn_nose_tail_jpn3 – determines nose and tail positoiin from tracking data

-fn_buildSIAGGmatrix – uses social interaction data and aggressive behavior data to create a matrix of temporally ordered behaviors

-**fn_buildSImatrix_nonsocialcontrol** - uses social interaction data and walk data to create a matrix of temporally ordered behaviors

-**fn_buildSImatrix_socialcontrol** - uses social interaction data and male investigate male data to create a matrix of temporally ordered behaviors

- fn_load_everything_to_make_life_easier – Neunuebel lab specific function to load in relevant data, most scripts call this function, but requires access to our Z drive

- fn_make_dom_output_from_chases_and_flees – Neunuebel lab specific function to calculate list of aggressive behaviors

-fn_calculate_dom_index_individual_mice_right_version – some scripts call this function to calculate the aggression score

-script_classifier_accuracy_figures – makes beeswarm plots that show each data point from a predictive classifier with 1000 iterations by loading in a saved structure with values

fn_distance_traveled: function to calculate total distance traveled during a selected period of time (could be the entire recording; could be during a particular behavior, etc)

script_AGGfeaturesclassifier – code for comparing features of aggressive behaviors between sequence types, including a classifier. Was not included in the paper.

fn_distancefromfemale – calculates distance between aggressor/aggressed and a female at specific points of the sequence. Requires tracking data, meters_2_pixels, and the frame raet.

fn_position_to_distance – uses xy position to calculate distance between 2 points. Can be used to calculate distance traveled, or distance between animals, or distance between an animal and a point in the arena.

Fn_updated_interpolation – uses fn_position_to_distance and interp1 (Matlab specific) functions to calculate distance with interpolation

inout_mw_group_front_of_ellipse – function that determines initiator of each social interaction, when given the tracking data used to calculate social interactions.

Script_createtemporaldynamicsmatrix_AGG – finds where sequences occurred to make a matrix of start and stop times

Script_getoverlapwithzone – draws a circle around a zone and uses a function to determine when the mouse overlaps with the zone

fn_circle_intersection – used to determine when a mouse overlaps with a predefined zone

fn_determineangle – calculates heading direction relative to another mouse

fn_distancefromfemale – calculates distance from a female at a specific point in a behavioral sequence

fn_findinitiatior – determine who initiated a social interaction

fn_findinitiator – determine who initiated a social interaction, regardless of social status

fn_josh_plot – make dot plots

fn_joshplotlineplotcombined – makes dot plots with lines that connect related data points

fn_outer_points – necessary for social interaction program

fn_plot_ellipse_jpn_4 – plot an ellipse around an animal

fnReadFrameFromVideo – find a specific video frame when given the file location of a .avi file

fnReadVideoInfo – get information about a video file

inout_mw – for social interaction program

load_track_file_jpn – generates relevant tracking information in pixels and meters

mouse_body_alt – allows you to plot the shape of a mouse

mouse_distance – tracking function

next_closest_mouse – tracking function

plot_axes - tracking function

polyplot – tracking function

prep_trajectory_jpn – tracking function

qinterp – tracking function

shadedErrorBar – makes shaded error bars

tconv – tracking function

rvel – tracking function

closest_mouse_investigate – finds closest mouse to extract investigations

clean_jaaba_indx – excludes events that don't meet behavioral criteria (JAABA)

fn_create_investitage_list – function to create a list of investigation events

assign_jaaba_investigate_output_jpn- calculates investigator for each relevant behavioral event

assign_jaaba_fight_output_jpn – assigns animals involved in a fight event

fn_create_fight_list – function to create a list of fights

assign_jaaba_flee_output_jpn- assigns animals involved in flees

fn_create_flee_list – function to create a list of fights

fn_create_walk_list – function to create a list of walks

fn_extract_events_from_list – uses information to extract relevant events

assign_jaaba_male_male_chase_output_jpn – assigns animals involved in chases

fn_create_male_male_chase_list –function to create list of chases

get_pos – function for calculating an animal's position

fn_find_dist_to_corner – used by some JAABA code to remove behaviors that occur too close to a corner
